# Dissection of genomic features and variations of three pathotypes of *Puccinia striiformis* through whole genome sequencing

**DOI:** 10.1038/srep42419

**Published:** 2017-02-17

**Authors:** Kanti Kiran, Hukam C. Rawal, Himanshu Dubey, R. Jaswal, Subhash C. Bhardwaj, P. Prasad, Dharam Pal, B. N. Devanna, Tilak R. Sharma

**Affiliations:** 1 ICAR- National Research Centre on Plant Biotechnology, Pusa Campus, New Delhi, India; 2 Indian Institute of Wheat and Barley Research, Regional Station Flowerdale, Shimla, H.P., India; 3 Indian Agricultural Research Institute, Regional Station, Shimla, H.P., India.

## Abstract

Stripe rust of wheat, caused by *Puccinia striiformis* f. sp. *tritici*, is one of the important diseases of wheat. We used NGS technologies to generate a draft genome sequence of two highly virulent (46S 119 and 31) and a least virulent (K) pathotypes of *P. striiformis* from the Indian subcontinent. We generated ~24,000–32,000 sequence contigs (N50;7.4–9.2 kb), which accounted for ~86X–105X sequence depth coverage with an estimated genome size of these pathotypes ranging from 66.2–70.2 Mb. A genome-wide analysis revealed that pathotype 46S 119 might be highly evolved among the three pathotypes in terms of year of detection and prevalence. SNP analysis revealed that ~47% of the gene sets are affected by nonsynonymous mutations. The extracellular secreted (ES) proteins presumably are well conserved among the three pathotypes, and perhaps purifying selection has an important role in differentiating pathotype 46S 119 from pathotypes K and 31. In the present study, we decoded the genomes of three pathotypes, with 81% of the total annotated genes being successfully assigned functional roles. Besides the identification of secretory genes, genes essential for pathogen-host interactions shall prove this study as a huge genomic resource for the management of this disease using host resistance.

Stripe or yellow rust of wheat has been reported in more than 60 countries in the world^1^. The stripe rust disease is caused by the biotrophic fungi *Puccinia striiformis* f. sp. *tritici (P. striiformis*)^2^,^3^,^4^. Yield losses caused by stripe rust disease have ranged from 10% to 70% depending on the disease response of the cultivar in most of the wheat-growing areas. Infection at very early stages of wheat growth as well as its subsequent persistence during the growing season can cause up to 100% losses^1^. *P. striiformis*-infecting wheat is further classified into different pathotypes based on their differential response on a standard set of wheat lines. Based on these standard sets, Allison and Isenbeck (1930), for the first time, established the existence of different pathotypes in *P. striiformis*^5^. New pathotypes of yellow rusts emerge very quickly, in India, eight new pathotypes have been reported in the last 20 years. Recently, *Yr9 vir*.(46S 119), a new pathotype of yellow rust pathogen, has emerged and spread into the northwestern plains of India, where traditionally a majority of the areas under wheat cultivation are occupied by wheat varieties containing the rust resistance gene *Yr9*^6^. Yellow rust pathogen uses different modes of dispersal of spores for successful infection and spread between and within different geographical areas^7^. Stepwise range expansion is yet another major mode of dispersal that typically occurs over shorter distances, within a country or a region. A good example of this type of dispersal mechanism includes the spread of a*Yr9*-virulent pathotype of *P. striiformis* that originally evolved in eastern Africa and migrated to South Asia through the Middle East and West Asia in a stepwise manner over a period of ~12 years and has lead severe epidemics along its path^8^.

Molecular studies involving stripe rust for functional characterizations of fungal genes have been extremely difficult, as this fungus cannot be cultured on artificial media^9^,^10^. The published draft sequence of *P. striiformis* 130 (64.8 Mb) with 22,185 annotated protein-coding genes^11^ and the draft sequence of Chinese isolate CYR32 (110 Mb assembly) with 25,288 protein-coding genes by Zheng *et al*.^12^ are important genomic resources to be used to identify secreted effector proteins. Recently, Hubbard *et al*.^13^ through transcriptome sequencing have described the recent dramatic shift of *P. striiformis* populations of the United Kingdom, unraveling the diverse population of this pathogen^13^. Additionally only couple of published reports about *P. graminis*^14^ and *P. triticina*^15^,^16^ are available. Thus, multiple whole-genome sequencing and resequencing strategies of this pathogen have provided an opportunity to characterize pathogen populations at a more precise and accurate level on a much wider scale. Furthermore, it has also helped in the analysis of repeat elements and large-scale divergence in genomic data, leading to the proper understanding of evolution of rust fungi. A wide range of methods for estimating diversification selection are now available, and signatures of diversifying selection have been predicted computationally in several filamentous plant pathogen effectors^17^,^18^. Therefore, such in-depth genome-wide studies would be beneficial for the development and deployment of yellow rust-resistant wheat cultivars.

Genome-wide studies on plant pathogens have widely focused on identifying effector proteins during the early stages of infection. Before or during infection, the expression of small secreted proteins with high sequence divergence has been reported^19^,^20^,^21^,^22^,^23^. Effector proteins with RXLR motif are among the most well-defined conserved motifs studied in oomycetes and are thought to be responsible for the translocation of fungal effectors into the host cells in the absence of pathogen^24^,^25^,^26^,^27^. It has been reported that fungal effectors of rust fungi lack such ubiquitous effector motifs unlike the oomycete fungi^28^,^29^. Although, previous studies have reported a rust pathogen and an ectomycorrhizal fungus along with both oomycete and other fungi, to contain functional RXLR variants, which act as a mediator for their transduction into plant cells in the absence of the pathogen^25^,^26^,^30^,^31^. Gu *et al*.^27^ have also reported a predicted effector from *P. striiformis* f. sp. *tritici* (GenBank accession number ES322018.1), which can be secreted and can enter plant cells via a conserved RXLR-like motif KRLTG. They also reported that the homologs of this protein are conserved across other fungal plant pathogens, including *Puccinia graminis* f. sp. *tritici, Melampsoralini, Melampsora laricispopulina, Magnaporthe oryzae, Alternaria brassicicola*, and *Pyrenophora tritici-repentis*, further suggesting an important role of this protein in biotrophic and necrotropic plant pathogen biology and/or pathogenesis.

All the epidemiological and evolutionary processes that determine the patterns of disease occurrence and prevalence mostly depend on the genotypic interactions between the pathogen and the host and are central to the variation within and among pathogen populations. However, there are different mechanisms whereby individual pathogen lineages or species may gain variation and evolve under natural conditions. In this study, we sequenced the genomes of three *P. striiformis* pathotypes (46S 119, 31 and K) from India, which are phenotypically and phylogenetically distinct from one another. This is the first report on the whole-genome analysis of *P. striiformis* pathotypes from India, identified from different epidemiological regions. The objectives of the present study are (i) to generate a high-quality draft genome sequence of a highly virulent pathotype (46S 119) along with two other pathotypes (31 and K) of *P. striiformis*, (ii) to perform genome wide analysis across the three pathotypes, and (iii) to understand molecular basis of variation in this fungus.

## Materials and Methods

### Materials used

Three *P. striiformis* pathotypes from India with distinct virulence profiles were selected for genome sequencing (Table 1). The virulence profiles of the three pathotypes, viz. 31 (67S64), K (47S102), and *Yr9 vir*. (46S 119), used in the study were confirmed on different sets of wheat differential hosts containing *Yr* resistance genes and were maintained at the Regional Station, Indian Institute of Wheat and Barley Research (Flowerdale, Shimla, India). Pathotype 31 is the least virulent among the three and is avirulent to rust resistance genes *YrA, Yr3, Yr4*, and *Yr9*, whereas pathotypes K and 46S 119 are virulent to all these genes, with an exception of pathotype K being avirulent on *Yr9*^32^,^33^. The urediniospores of these pathotypes were maintained at a susceptible wheat genotype Agra local from a single spore infection.

### Genomic DNA isolation

Genomic DNA was isolated from the urediniospores of *P. striiformis* pathotypes 31, K, and 46S 119 with slight modifications^34^. Thirty milligrams of dried urediniospores were ground to fine powder in liquid nitrogen using mortar and pestle. Then, 550 μl of extraction buffer [100 mMTris-HCl (pH 8.0), 20 mM EDTA (pH 8.0), 1.4 Mm NaCl, 2% cetyltrimethylammonium bromide (CTAB)] was added to the fine powder of spores and transferred to a 1.5 ml microcentrifuge tube. Proteinase K (Fermentas, USA) was added to a final concentration of 0.2 mg/μl and the tube was incubated for 2 h at 65 °C. Denatured proteins were removed once by extraction with 600 μl Tris saturated phenol/chloroform/isoamyl alcohol (25:24:1, v/v/v) and then by repeated extractions with 600 μl Tris saturated chloroform/isoamyl alcohol (24:1, v/v). After centrifugation, the aqueous phase was removed and DNA was precipitated with 1/10th volume of sodium acetate (3 M; pH 5.3) and double volume of absolute alcohol. DNA was pelleted, dried, and resuspended in 40 μl Tris-EDTA buffer [10 mMTris-HCl (pH 7.5), 1 mM EDTA]. An aliquot of the extracted DNA was separated by electrophoresis on 1% agarose gels for visualization and quantification.

### Generation of genome sequence and assembly

Paired end libraries from the genomic DNA of all the three pathotypes were separately prepared with 100 bp paired end sequenced data using Hiseq1000 (Illumina) automated sequencer (Illumina, Inc., San Diego, CA, USA). The genome sequences (100 bp on average) were aligned against the *P. striiformis* pathotype 78-1 (Puccinia Group Sequencing Project, Broad Institute of Harvard and MIT (http//www.broadinstitute.org)^15^, using ABySS software. Reference-based assembly was performed for the processed data by GS Reference Mapper (Roche) with default parameters (minimum read length = 20 bp, minimum overlap length = 40 bp, minimum overlap identity = 90%, alignment identity score = 2, and all contig threshold = 100) with the genome sequence of *P. striiformis* pathotype 78-1 as the reference. Raw reads of pathotype 31, K, and 46S 119 were also mapped against the assembled data of self and other two pathotypes. The quality of the assembly was carried out by QUAST 3.2 software tool (Fig. S1, Table S1). Furthermore, the *de novo* assembly of the unassembled reads as well as the alignment of the raw reads of each pathotype as a whole and individually with the assembled data of their respective partner was performed using CLC Genomics Workbench 7.0. with default parameters (minimum contig = 100 bp, 23 K-mer, similarity fraction = 80% and length fraction = 50%).

### Gene prediction and annotations

Genes were predicted from the large contigs (≥2 kb) by Ab initio gene prediction software FGENESH 3.1.2 (MolQuest2.2) with at least 80% homology against *Puccinia* spp. The software was trained on *Puccinia* matrix to predict genes from assembled contig sequences at Standard translation table. In-house developed PERL scripts were used to parse the FGENESH output and extract sequences. Predicted genes were BLAST searched against the National Center for Biotechnology Information (NCBI) EST data (for expression analysis. Genes (≥450 bases) were BLAST searched against the NCBI nonredundant (nr) protein database for functional annotations. The genes with significant hits (*E* ≤ e^−10^) were then grouped into different functional categories.

### Identification of repeat elements within *P. striiformis* genomes

The repeat elements belonging to various classes including long terminal repeats (LTRs), non-LTRs, and DNA transposon elements were identified using MapRep (MolQuest2). Out of the total transposable elements (TEs) identified nucleotide sequences greater than 200 bp for two major groups (i.e. Gypsy and Copia) were extracted from their respective contigs. The annotation of these elements was done by BLAST search of the FASTA files against the publicly available repeat database of fungi on Repbase (http://www.girinst.org/repbase/update/). To identify full-length LTR elements, the LTR_FINDER software tool^35^ (http://tlife.fudan.edu.cn/ltr_finder/) was used with all specific parameters checked and set against the *Saccharomyces* repeat inbuilt database. Tandem repeat sequences were detected with the Tandem Repeats Finder 4 software with default parameters^36^ (https://tandem.bu.edu/trf/trf.html). The SSR identification was done in all three genomes using MISA software and categorized using standard parameters (http://pgrc.ipk-gatersleben.de/misa/).

### SNP analysis

SNPs were detected using Sequence Alignment/Map tools (SAM tools) software package at 10X coverage with the quality value of Phred score ≥20. The SAM files generated by BWA was converted to bam file and processed by mpileup utility of SAM tool to generate a pile-up of readbases using the alignments to the reference sequence for the prediction of SNPs. Additionally, SNPs dectectionby CLC Genomics Workbench 7.0 was also performed (parameters, Ploidy = 2, Coverage 10–100,000, Variant Frequency ≥35%). The annotation of the SNPs (SAM tools) was performed using SnpEff software^37^ by using default parameters.

### Analysis for whole-genome segmental duplication (SD)

The whole-genome assembly comparison (WGAC) method^38^ based strategy was used to detect SDs. Self-BLAST search was done for all the assembled contig sequences within each genome to identify the possible SDs (sequences with at least 90% identity over >1000 bp alignment length or more). Self-hits, duplicate entries, and partial and reverse BLAST hits were removed to obtain the final list and the amount of SDs in the genome. Sequences of SDs were extracted from whole assembly using PERL scripts and then subjected to FGENESH and MapRep of MolQuest2.2 software package for the prediction of genes and TEs, respectively. Predicted genes were self-BLAST searched and parsed to remove duplicates and partial and redundant genes. Genes were BLAST searched against the nr database of the NCBI for functional annotation.

### Putative evidence of genetic recombination

Three largest genes in the SD regions of each of the three pathotypes were aligned by ClustalW and subjected to analysis using TOPALi (version 2.5) for detecting any possible events of genetic recombination by ‘Difference of Sum of Squares’ (DSS) method (McGuire and Wright, 2000)^39^ with window size of 500 and Jukes-Cantor model of nucleotide substitution. Prediction of putative recombination breakpoints were made by plotting the difference between SS_L_ and SS_R_ (DSS statistic) against the window centre where, SS_L_ and SS_R,_ are the sum of squares between the observed distances and the distances based on the tree for left-hand window and the right-hand window, respectively. Additionally, possible evidence of recombination was also identified among the largest predicted gene within the genomes of three pathotypes. Respective contigs bearing the gene were first aligned by Mauve software followed by the alignment of these genes by ClustalW. For *in silico* analysis default parameters of all the software were used in this study.

### Secretome analysis

Combinations of different software (SignalP version 4.1; www.cbs.dtu.dk/services/SignalP;TargetP version1.1, www.cbs.dtu.dk/ services/TargetP andPhobious, phobias, sbc.su.se/data.html) were used to define the secretomes of *P. striiformis* pathotypes (Fig. S2). Initially, proteins (>30 amino acids) with a SignalP D-score = Y and a cut off value, 0.45 for 0 Tm/0.50 for 0.50 Tm and TargetPLoc = S were combined. These were then scanned for transmembrane spanning regions using TMHMM (version2.0; http://www.cbs.dtu.dk/services/TMHMM). Peptides with 0 or 1 transmembrane regions were retained and transmembrane region located in less than 10 amino acids in mature peptide from predicted cleavage site as well as proteins with highly probable GPI-anchor predicted by predGPI (http://gpcr.biocomp.unibo.it/predgpi/pred.htm) were taken for further analysis. The eventual locations of these proteins were predicted by the integral prediction of protein location score obtained by ProtComp version 10 (http://linux1.softberry.com/berry.phtml/berry.phtml?topic=protcompan&group=programs&subgroup=proloc) Proteins showing the integral prediction of protein location and extracellular secreted (ES) and mature peptide length (after trimming sequence based on cleavage site predicted by SignalP and TargetP) of more than 20 amino acids were kept in a final secretome data set. WoLF PSORT analysis was performed using “run WoLfPsort Summary fungi” to find peptides with a high probability of secretion using WoLF PSORT version 0.2 (http://www.wolfpsort.org/WoLFPSORT_package/version0.2). BlastP was used for the annotation of predicted secretome. Conserved domains in the secretome were predicted through the conserved domain database at the NCBI (http://www.ncbi.nlm.nih.gov/Structure/cdd/wrpsb.cgi) using E-value threshold of 0.01A calculation of cysteine content in ES proteins was performed on a mature peptide sequence after removing the predicted signal peptide. The number of cysteine residues in mature peptide was counted and divided by the total number of residues in mature peptide and converted to percentage. Conserved domain identification was performed using Pfam database (Pfam-A version 27) with profile gathering cutoff threshold.

### Diversifying selection analysis in extracellular secreted proteins

Orthologous ES proteins among *P. striiformis* pathotypes were found using OrthMcl version 1.4 using default parameters (–mode 1, *P* = 1e^−5^). Clusters having at least one representative gene sequence from each of the *P. striiformis* genomes were separated for further analysis. Sequences from this cluster with genes having methionine (M) as a first codon and having length at least 100 amino acids were considered for diversifying selection analysis. Ortholog sequences in each cluster were aligned by ClustalX version 2.0^40^. The format of the produced alignment files was converted to PAML by PAL2NAL software version 14.0^41^. YN00 of pamlX version 1.3.1^42^ was used to estimate nonsynonymous and synonymous substitution rates (pairwise *dN*/*dS* ratios) for genes having at least one ortholog. For genes possessing at least two orthologs, the pairwise mean of *dN*/*dS* ratios was calculated and site-specific diversifying selection was additionally performed using CODEML of pamlX version 1.3.1. Two likelihood ratio tests (LRTs) for site-specific diversifying selection were used: model M1 (neutral) to model M2 (selection) and model M7 (β) to model M8 (β and ω). The significance of LRTs was assessed using *χ*^2^ tests at the significance threshold of *P* < 0.05. Site-specific diversifying selection for genes was considered true if both M1/M2 and M7/M8 LRTs were found to be significant^29^.

### *In silico* identification of pathogenicity-related genes in *P. striiformis* pathotypes

The predicted genes in each genome were BLAST searched against 2647 protein sequences of PHI-base (Pathogen-Host Interactions database version 3.6; http://www.phi-base.org/). Genes with significant hits (with *E* ≤ e^−20^ and bit score ≥100) against PHI were considered as the pathogenicity-related genes.

### Evolutionary analysis of *P. striiformis* genomes

A significant rearrangement of fragments or genes may occur eventually during the course of evolution; therefore, traditional multiple sequence alignment on complete genome sequences cannot be used^43^,^44^. Progressive Mauve version 20150226 build 10^45^ with default parameters was used to perform comparative evolutionary analysis based on whole-genome alignment. Besides the three pathotypes used in this study (31, K, and 46S 119), seven other published genomes of *P. striiformis* from different geographical origins were considered for analysis (Table S2). Comparative analysis was performed on all 10 genomes by studying the neighbor joining tree produced as described by Saitou and Nei (1987)^46^.

## Results

### Genome sequencing and assembly

Genomes of three pathotypes 31, K, and 46S 119 of the fungal wheat rust pathogen *P. striiformis* were sequenced using whole-genome shotgun sequencing approach with 100 bp (Illumina HiSeq-1000) paired end reads, generating a total of 6.1 to 8.6 Gb sequence data. These data were subjected to the quality assessment by aligning the reads from each strain with the data of *P. striiformis* 78-1 (Puccinia Group Sequencing Project, Broad Institute of Harvard and MIT (http//www.broadinstitute.org)^15^. On average, ~83% to 88.5% genome breadth coverage (assembled genome size/target genome size×100) was obtained for each of the three pathotypes. Furthermore, pathotype 78-1 was again used as a reference sequence to assemble genomes of the three pathotypes using GS Reference Mapper Software (version 2.0 Roche) and a total of 70% to 79% reads were mapped to the reference sequence. This resulted in ~24,000 to 32,000 sequence contigs (N50; 7.4–9.2 kb), which has ~86X to 105X read depth coverage of *P. striiformis* genomes with an assembled genome size ranging from 66.2 to 70.2 Mb (Table 2). An estimate of the quality of assembly was carried by QUAST 3.2 software tool (Fig. S1, Table S1). Approximately 12 kb (31), 11.5 kb (K), and 4 kb (46S 119) data were predicted as misassembled by the software tool (Table S1). The analysis of the reads that did not assemble with the reference revealed that these reads were a mixture of the reads containing short-sized reads, repetitive (single nucleotide, dinucleotide) sequences. Some of the sequences otherwise looked fine and were cross-checked for possible contaminations. Therefore, these unmapped reads were further assembled *de novo* for investigating if the data contained isolate-specific genes (Table S3).

Raw reads of each pathotype were further mapped against the assembled data of their respective pathotype used as a reference, which resulted in 69% to 78% of mapped reads that assembled into 66 to 70 Mb size (Table S3). Rust fungi urediniospores are asexual dikaryotic (two unfused, haploid nuclei in one cell) spores. Therefore, to evaluate the existing genetic variation between the two nuclei within the three *P. striiformis* pathotypes, the sequence read of a pathotype was aligned to the assembled contigs of the same pathotype. An SNP frequency of 5.30 ± 2.78 SNPs/kb (on average) was identified between the two nuclei within a single pathotype (intra-pathotype SNPs), (Table S4).

Additionally, to explore the diversity of *P. striiformis* across the three pathotypes, we aligned the reads of each pathotype to the assembled contigs of the other two pathotypes to find heterokaryotic and homokaryotic SNPs. On average, heterokaryotic SNPs across the three pathotypes were more frequent (4.67 ± 1.17 SNPs/kb) than homokaryotic SNPs (1.90 ± 1.27 SNPs/kb). The highest levels of diversity were found when reads of isolate 46S 119 were mapped onto the other two isolates with an average of 6.13 ± 0.13 SNPs/kb for heterokaryotic SNPs and 1.56 ± 0.12 SNPs/kb for homokaryotic SNPs. When the other two pathotypes (K and 31) were compared, the heterokaryotic SNP frequency was 4.0 ± 0.45 SNPs/kb and the homokaryotic SNP frequency was 0.68 ± 0.02 SNPs/kb (Table S5).

### Gene prediction and annotation

**Figure 1:**
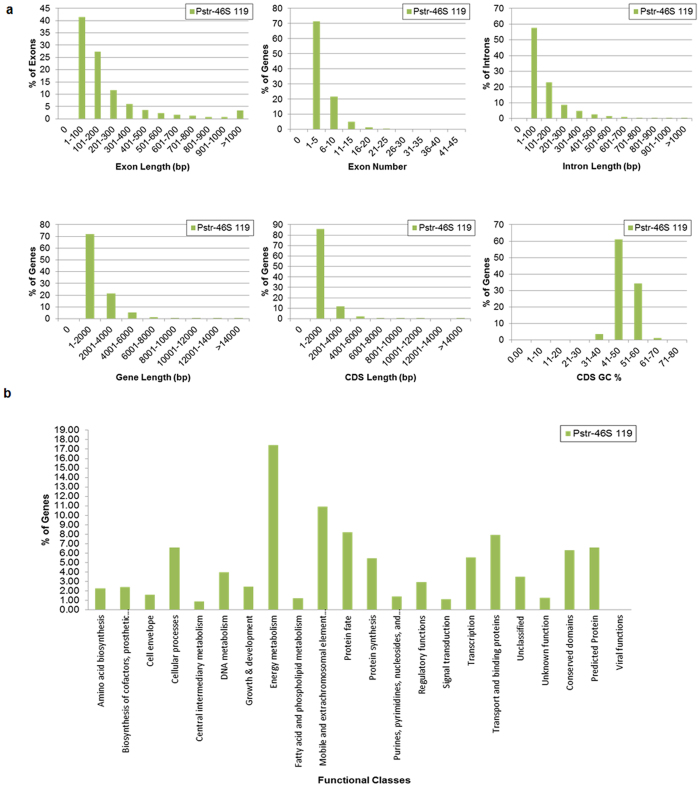
Gene prediction and annotation in the genomes of three pathotypes of *P. striiformis*. (**a**) Comparative validation of gene prediction performed with six different gene parameters among the genomes of the three pathotypes of *P. striiformis* including exon length, exon number, intron length, gene length, CDS length and GC% within CDS regions. (**b**) Annotation of the genes predicted in the three genomes showing percentage of genes categorised in different functional groups. Analysis of the genome sequence of pathotype 46S 119 is given for both comparative validations and annotations of genes. Similar analysis of pathotyppes 31 and K as is given in Supplementary Figures (Fig. S3a and S3b).

We predicted 18,362, 18,880, and 19,795 genes in pathotypes 31, K, and 46S 119, respectively, using (homology-based) FGENESH gene prediction software. The largest gene comprised 13,400 bp in pathotypes 31 and K, whereas, in pathotype 46S 119, it was 16,100 bp, which belongs to the family of dynein heavy-chain proteins having vital roles in biological processes, including ciliary beating, cell division, and intracellular transport^47^. Significant BLAST hits for genes ≥450 bp (12,354, 12,699, and 13,216) resulted in 10,082, 10,328, and 10,774 genes in pathotypes 31, K, and 46S 119, respectively. These genes accounted for more than 81% of the total annotated genes (only ≥450 bp) in all the three pathotypes out of which 57.4%, 57.3%, and 56.6% from 31, K, and 46S 119, respectively, were hypothetical (Table S6). This can be attributed to the less information of fungal rust genomes available in public databases. The remaining 19% genes in all the three pathotypes did not produce any significant hit against the nr database. The quality of gene prediction was assessed by comparing the length distribution of genes, CDS, exons, and introns and the distribution of exon number per *P. striiformis* gene among the three pathotypes. All the three genomes were found to be similar to each other with respect to all the major parameters analyzed, except for a higher percentage (57.5) of short introns observed in pathotype46S 119 and long introns (~7.3) observed in pathotypes31 and K (Fig. 1a; Fig. S3a). Furthermore, the categorization of the genes according to various functional classes resulted in a similar pattern of gene distribution among all the three *P. striiformis* genomes, but there was some specificity towards certain classes (Fig. 1b, Fig. S3b,S1 Notes, Table S7).

Gene predictions within the *de novo* assembled data of unmapped reads resulted in 11,333, 27,872, and 97,011 genes in pathotypes 31, K, and 46S 119, respectively. A BLAST search of these genes from unmapped reads showed no significant hits (BLAST hits with *E* ≤ e-10 and bit score ≥100) for 97% to 98% against the reference genome (pathotype 78-1). The inter-species BLAST search of these predicted genes also indicated that 88% to 97% of these genes could be pathotype specific with no significant hits in other two pathotypes. Pathotype 31 shared 128 similar genes (100% identity and same length) with pathotype K and 62 genes with 46S 119, whereas pathotypes K and 46S 119 shared 66 such genes with pathotype 31 (Tables S8 and S9).

**Figure 2:**
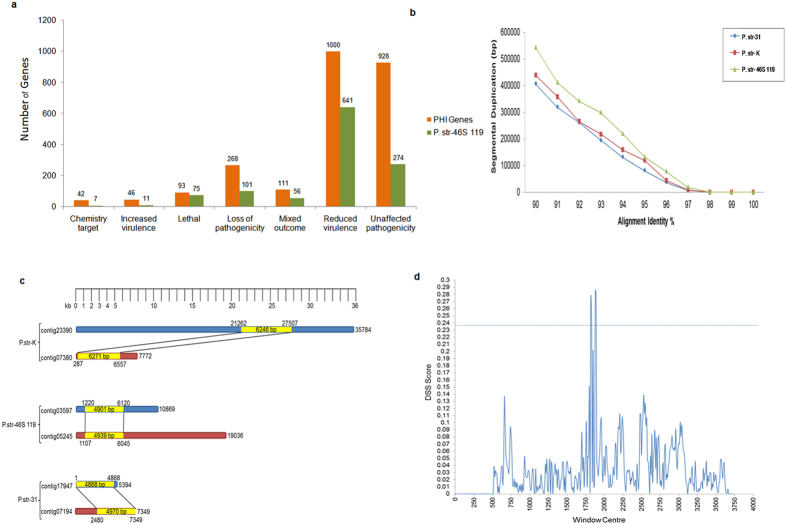
Analysis of pathogenecity genes and segmental duplication in the genomes. (**a**) Number of genes within pathotype 46S 119 distributed into various functional classes realted to pathogenicity. Analysis of genome sequence pathotypes 31 and K is given as Supplementary Figures (Fig. S4). (**b**) Segmental duplication observed within the three pathotypes in the scatter plot of percentage aligment identity vs length of sequences. (**c**) Examples of contigs showing regions of segmenteal duplicated regions within the three pathotypes. (**d**) Analysis of evidence of genetic recombination in the three largest genes within the SD regions of the three pathotypes. Putative recombination breakpoints were observed at positions 1820, 1830, 1880 and 1890. The horizontal line is the 95% significance point of DSS obtained by parametric bootstrapping. Plot representing difference of sums of squares (DSS) against the window centre of the alignment of genes.

Among all predicted *P. striiformis* genes, only 1130 (6.1%), 1158 (6.13%), and 1165 (5.89%) genes from pathotypes 31, K, and 46S 119, respectively, had homologues with known functional genes in the PHI database (Table S10), and the majority of these homologues belong to reduced virulence (Fig. 2b, Fig. S4).

### Identification of duplicated regions in *P. striiformis* genomes

A well-established WGAC method-based approach was used to identify SDs (i.e. blocks of sequences having ≥90% sequence identity with ≥1 kb alignment length) in the assembled genomes by self-BLAST search of the assembled contigs of each of the three *P. striiformis* genomes. The maximum amounts of SDs were identified in pathotype 46S 119 with 2.89% (2.03 Mb) followed by 2.30% (1.61 Mb) and 2.15% (1.43 Mb) in pathotypes K and 31, respectively (Table S11). A decline in the SD sequence length with the increasing percentages ranging from 90% to 97% was observed in all the three pathotypes, when an individual percentage of alignment identity against the length of sequences was considered (Fig. 2b and c). At an individual genome level, pathotype 46S 119 had a higher level of duplication than the other two pathotypes. However, the analysis for large blocks (>5 kb) revealed that all three genomes were poor in these blocks, as we could find just one such SD block (6.2 kb) in pathotype K. Furthermore, among the observed SDs, there were only 9% to 11% of SD blocks falling under high-identity duplications class (identity >94%). Besides, possible evidences of recombination events were analyzed by two methods involving two datasets. Three largest genes within the SD regions of the pathotypes were analysed by the software TOPALi version 2.5. Four large peaks at positions 1820, 1830, 1880 and 1890 were obtained above the threshold (95% significance point of DSS score) demonstrated the possible recombination breakpoints (Fig. 2d). Additionally an alignment based method involving Mauve and ClustalW on the largest predicted gene (dynein heavy chain) from all the three pathotypes was analysed for variations and a possible recombination event within the Site specific recombination by insertion mechanism have been reported earlier^48^,^49^,^50^,^51^. From the whole genome alignment of the assembled contigs of the three pathotypes by MAUVE, randomly a region with some visual variation was deduced. Analysis of the region within the respective contig positions revealed that the region was encoded by dynein heavy chain gene, the largest gene identified in all the pathotypes. Nucleotide alignment of the genes showed an extra highly repetitive segment of 2751 bp at the start position in pathotype 46S 119. The gene was well conserved in all the three pathotypes irrespective of small gaps at some positions in pathotypes K and 31. Some extra nucleotide insertions in pathotype 46S 119was finally confirmed by alignment of the protein sequences as well. The pathotype 31 and K were 99.8% similar and pathotype 46S 119 was 52% similar to 31 and K. The results suggest the possibility of an insertional mechanism by site specific recombination event within the gene (Fig. S5).

### Repetitive sequences in *P. striiformis* genome

**Figure 3:**
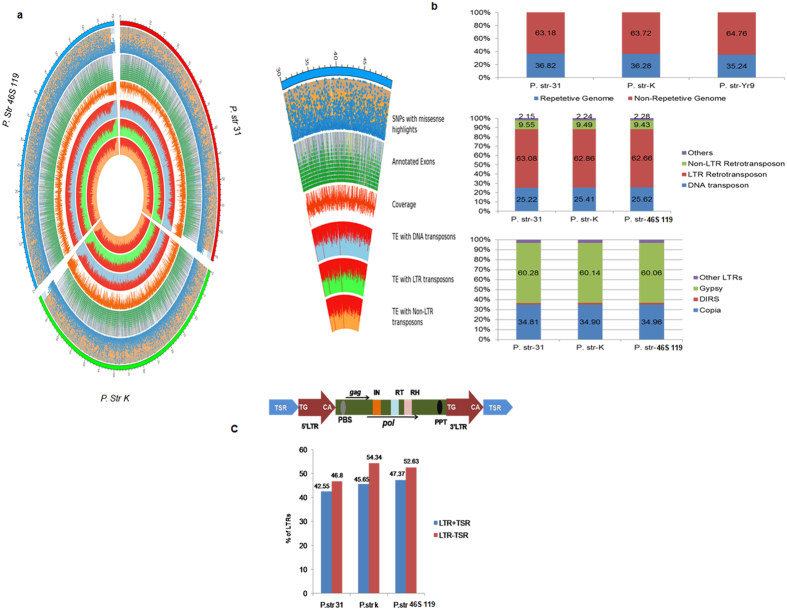
Genome wide analysis of various genomic features of *P. striiformis* pathotypes. (**a**) CIRCOS plot of three individual *P. str* pathotypes depicted outside the outer most circle with 1 Mb breakpoints increasing in the clockwise direction and covering the whole genome size. The outermost circle is the density scatter plot of SNPs (blue color) highlighting missense SNPs in orange colour and Nonsense SNPs in white colour. The second inner circle is a density tile plot of all the annotated exons (green colour) highlighting five major categories of genes namely cellular processes (orange), DNA metabolism (yellow) energy metabolism (blue) mobile -extra chromosomal elements (purple) and transport and binding proteins (grey). Next inner circle is the density histogram plot depicting total genome coverage (red colour). The inner most three circles (red colours) are density histogram plots of total repeat (TE) element contents with blue colour highlighting the DNA transposons (outer circle) followed by LTR elements highlighting in green colour (middle circle) and the Non-LTR elements highlighting in orange colour (inner most circle). (**b**) Histogram plots of the percentage distribution of repetitive and non-repetitive content in the genomes of the three pathotypes followed by the percentage distribution of TE content within their genomes categorized into three major groups of DNA transposons, LTR and Non LTR elements, and percentage distribution of Gypsy, Copia and DIRS and other Sub groups within the LTR elements. The results for the same were obtained by detailed genome wide analysis performed through various software tools. (**c**) Figure of a full length intact LTR elements found in fungal genomes followed by percentage of intact LTR elements found in the three pathotypes (31, K and 46S 119) with TSR borders (blue bars) and elements lacking TSR borders (red bars).

The total repeat content (TEs) identified in the three *P. striiformis* genomes accounted for ~36% (Table S11). The majority of the repeats (>70%) were retrotransposons, 25% of the elements accounted for DNA transposons, and the remaining ~2% were unclassified elements. Among retrotransposons, the LTR family was the most abundant (63%) in all the three genomes, out of which Copia (34%) and Gypsy (60%) were the two most abundant subfamilies with a minimal percentage of DIRS elements (Fig. 3b). To investigate whether, the portions of the unclassified repetitive sequences are *P. Striiformis* pathotype specific or belong to some novel class, all contig files of the genomes were subjected to the analysis with LTR_FINDER. We obtained 43, 46, and 57 full-length LTR elements in the genomes of 31, K, and 46S 119, respectively (Table S12). The full-length elements with left and right target site repeats (TSR) along with 3′ and 5′ LTR sequences corresponded to 42.5%, 45.6%, and 47.3%, respectively, whereas LTR elements lacking TSRs but having 3′ and 5′ LTR sequences corresponded to 46.8%, 54.3%, and 52.6%, respectively (Fig. 3c). These elements despite being full-length either contained truncated internal protein coding sequences or lacked some of the essential protein domains, which could be classified into a functional category.

### Identification of SNPs and InDels

**Figure 4:**
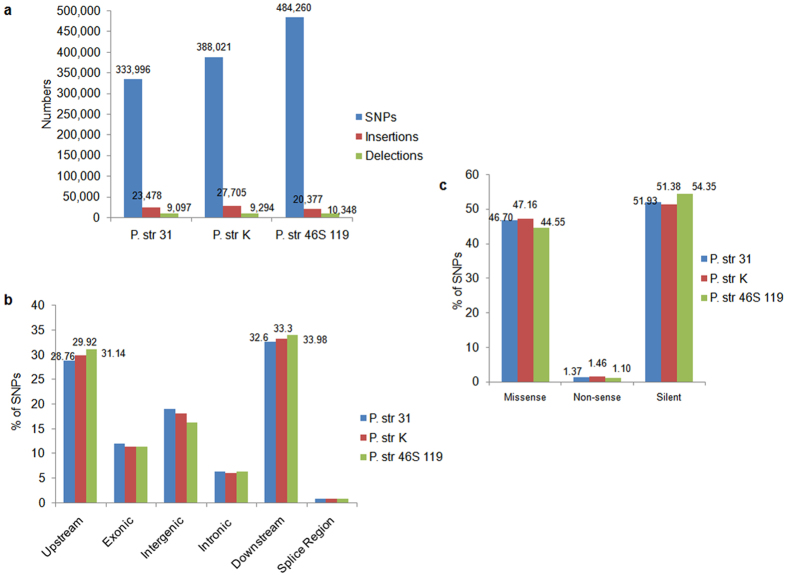
Genome wide SNP analysis within the genomes of three pathotypes. (**a**) Identification of all mutations within the genome of *P. striiformis* pathotypes (**b**,**c**). Genome wide percentage distribution of SNPs within different genomic regions including types of SNPs in the respective genomes of the three pathotypes of *P. striiformis*, respectively.

SNPs and Indels were identified by two individual software tools (samtools.sourceforge.net/mpileup.shtm and CLC workbench 7.0). Both the software produced a fairly similar pattern of the identified mutational events in the three pathotypes. A total of 3.3 million (pathotype 31), 3.9 million (pathotype K), and 4.8 million (pathotype 46S 119) SNPs were predicted via multiple stringent filtering criteria by Samtools. Simultaneously, 3.1, 2.9 and 4.6 million SNPs were predicted by CLC Genomics workbench 7.0 in pathotypes 31, K and 46S 119, respectively. Overall, insertions (average = 23,853 by samtools and average = 34,302 by CLC) were more than deletions (average = 9579 by samtools and average = 13,093 by CLC) as observed collectively as well as individually in all three pathotypes. Eventually, the results produced by samtools were considered for indepth study of SNPs in the three pathotypes. A significant role of SNPs compared to InDels in shaping the genomes was reflected in the three pathotypes (Fig. 4a). SNP distribution pattern revealed that 86.2% SNPs in pathotype 31 and 87.8% SNPs in both pathotypes K and 46S 119 belonged to the nongenic region (intergenic, intronic, upstream and downstream untranslated, splice region), whereas 12.8% of SNPs in pathotype 31 and 12.2% of SNPs in both pathotypes K and 46S 119 were in the genic region. The 5′ and 3′ untranslated regions SNPs accounted for ~30% to 33% in these three pathotypes but with a difference of ~3% between pathotype 46S 119 and 31 (Fig. 4b). On average, a total of 127,502 SNPs (~12%) in the exonic regions comprising ~57,730 (46.1%) missense, 1617 (1.30%) nonsense, and 66,241 (52.6%) silent mutations were classified as coding sequence variants. It indicated that, on average, ~47% of the gene set are affected by nonsynonymous substitutions in the three *P. striiformis* genomes (Fig. 4c)). The differences recorded were relative to the reference genome (Race 78-1) used in the study.

### Identification of ES proteins in *P. striiformis* genomes

**Figure 5:**
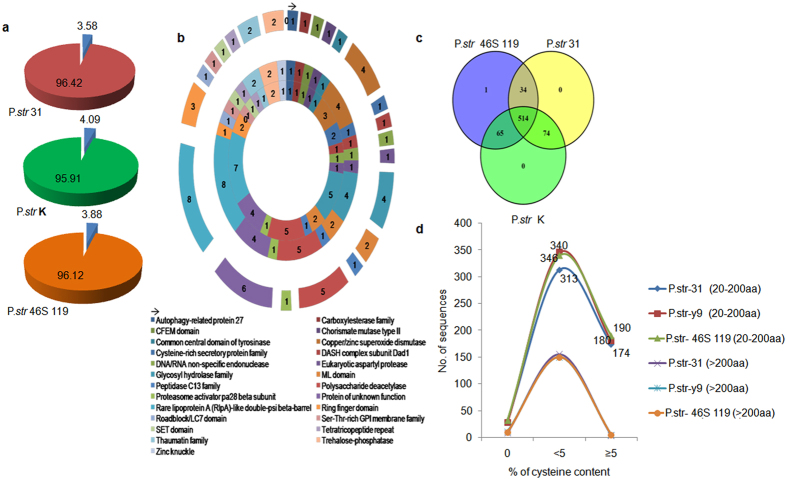
Genome wide secretome analysis of the three pathotypes of *P. striiformis*. Whole genome secretory proteins of three pathotypes (*P. str*31, *P. str* K and *P. str* 46S 119) were identified through various software (Tables S13 and S14). (**a**) Pie chart depicting percentage of extracellular proteins (Blue) and % of total secretory proteins (red, green, orange) in all the three pathotypes. (**b**) Functional domains identified within the extracellular proteins in these three pathotypes obtained through Pfam database with the inner most circle representing pathotype *P. str* 31 followed by pathotype *P. str* K (middle circle) and the outer most circle showing pathotype*P. str*46S 119. (**c**) Venn diagram representing comparative analysis of the extracellular proteins based on homology within and among the three pathotypes. (**d**) Extracellular proteins <200 a.a. searched for cysteine content showing pathotype 46S 119 to possess more sequences with higher percentage of cysteine than pathotype K and pathotype 31.

Of the 17,280, 17,750, and 18,561 proteins (>30 amino acids) encoded in pathotypes 31, K, and 46S 119, respectively, we could annotate a total of 1751, 1811, and 1809 proteins as classical secretory proteins by SignalP version 4.1. The total proteins were also analyzed by TargetP version 1.1 (2880, 2986, and 3071 secretory proteins in the pathotypes 31, K, and 46S 119, respectively) and with Phobius^52^, standalone perl script version (Fig. S2). After merging the filtered sets (SignalIP and TargetP) and the removal of duplicate segments, proteins were then scanned using TMHMM software, leading to the prediction of 322, 370, and 353 transmembrane proteins in pathotypes 31, K, and 46S 119, respectively. These transmembrane proteins were removed from the protein data set. Finally, a total of 687, 727, and 720 sequences were predicted as ES proteins with only mature peptide sequences of more than 20 amino acids as analyzed by ProtComp version (Table S13). These ES proteins represent ~4.0% of the total predicted proteins of the three *P. striiformis* genomes (Fig. 5a). ES proteins predicted in the previous step were further screened using WoLF PSORT version 3, resulting in 211, 200, and 213 sequences as subcellularly localized within their respective genomes (Table S14).

### Annotation of *P. striiformis* secretome

Of the 687, 727, and 720 ES proteins identified by ProtComp version 10 in pathotypes 31, K, and 46S 119, respectively, 98 (14.3%), 95 (13.1%), and 95 (13.1%) proteins showed significant BLASTP matches with proteins deposited in the nr database, and 89 (12.9%), 86 (11.8%), and 83 (11.5%) proteins represented significant BLASTP matches with hypothetical protein homologs. Conserved domains with precise function were searched with Pfam (Fig. 5b). These corresponding proteins could be identified as novel targets in the three pathotypes. Protein homologs with a precise functional description in the three pathotypes were far too less with only 8 proteins each in pathotypes 31 and K and 10 proteins in pathotype 46S 119 (Table S15). Out of these, four proteins, namely, α-galactosidase, glyceraldehyde-3-phosphate dehydrogenase, hAT family dimerization domain-containing protein, sterol 24-C-methyltransferase, were specific to pathotype 46S 119. Differentiation-related protein 1 and plasma membrane proteolipid 3 were specific to pathotype 31 and only one protein, ubiquitin-activating enzyme E1, was specific to pathotype K.

### Analysis of orthologs identified among *P. striiformis* pathotypes

Orthologous extracellular proteins among the three *P. striiformis* pathotypes were identified using OrthMcl version 1.4 with default parameters (–mode 1, *P* = 1e^−5^). It uses Markov cluster algorithm to group sequences, with inflation (−F) controlling cluster granularity, as described by Li *et al*.^53^. A total of 688 clusters were formed and then were separated into three major categories based on the three pathotypes involved in the analysis. Category 1 consisted of 514 clusters having at least one representative sequence ortholog from each of the *P. striiformis* genomes. Category 2 consisted of 34 clusters of shared orthologs between pathotypes 46S 119 and 31, 74 clusters of shared orthologs between pathotypes K and 31, and 65 clusters of shared orthologs between pathotypes 46S 119 and K. The third category had a cluster with a single copy of gene specific to pathotype 46S 119. Furthermore, pathotypes K and 31 did not show any copy of specific genes (Fig. 5c, Table S16). These results indicate that the genes are being commonly shared between pathotypes K and 46S 119 and between pathotypes 31 and K and vice versa but not between pathotypes 31 and 46S 119.

### Analysis of small cysteine-rich (SCR) ES proteins

Recent reports have shown that SCR secretory proteins suppress plant defenses to facilitate infection by manipulating host cell structure and function to obtain nutrients, especially in biotrophic fungi^54^,^55^,^56^,^57^,^58^. Although larger proteins can also act as effector proteins^59^, smaller proteins rich in cysteine were found to be mostly less than 300 amino acids as reported in several studies^14^,^60^,^61^,^62^,^63^. Also, recently, some of the identified and characterized proteins were less than 200 amino acids in length. Therefore, based on these studies, to analyze SCR proteins present in the three pathotypes within the total secretory proteins identified, we predicted 518, 554, and 562 SCR secretory proteins (20–200 amino acids) in pathotypes 31, 46S 119, and K, respectively. Among these, proteins rich in ≥5% cysteine residues corresponded to 193 (37.26%), 199 (37.80%), and 216 (38.2%) of the total SCR proteins in the three pathotypes, respectively, whereas, at ≥8% cysteine threshold, 19 (~3.6%) proteins each were identified in pathotypes 31 and 46S 119 and 26 (4.6%) proteins were identified in pathotype K. All these proteins were either hypothetical or unannotated (nonsignificant BLAST hits; Fig. 5d).

### Diversifying selection analysis of ES protein in *P. striiformis*

To identify the undergoing strong evolutionary pressure exclusively in genes that are pathogen associated within the three pathotypes, we performed the diversifying selection analysis of the orthologous ES protein coding genes. We used two methods from PAML software^64^. YN00 tool based on counting method, as described by Yang and Nielsen^65^, was used to estimate pairwise (nonsynonymous/synonymous) *dN*/*dS* ratios for all the ES genes that have at least one ortholog. Genes with at least two orthologs were further analyzed by CODEML for site-specific diversifying selection using two LRTs followed by assessing significance using *χ*^2^ tests. The *dN*/*dS* ratios were calculated for 73.98%, 99.12%, and 70.15% ES protein coding genes in the case of pathotypes 31, K, and 46S 119, respectively. The mean *dN*/*dS* ratio (0.12) was highest in pathotype 46S 119 than the other two pathotypes, although the number of genes analyzed in this pathotypewas lowest. The site-specific diversifying selection was performed for 59.27% of ES genes in pathotype31, 81.10% in pathotype K, and 59.27% in pathotype 46S 119 (Table S17). Potential genes analyzed to be undergoing site-specific diversity selection were similar in the three pathotypes (Table S18). Most of the genes either did not produce significant BLAST hits against nr database or were hypothetical genes, with a single gene found in all the three pathotypes to be recognized as putative f5 8 type c domain proteins (Tables S19 and S20).

### Identification of ES genes with *dN*/*dS* > 1 in *P. striiformis* pathotypes

Like most organisms, plant pathogenic fungi rely on mutation and recombination as the main sources of genetic diversification. The mechanisms of molecular evolution of *P. striiformis* genes and gene families are largely unknown. Nevertheless, it is expected that genes that need to adapt to the host and its defense mechanisms or that need to avoid host recognition are under severe diversifying selection. We examined extracellular genes with at least one ortholog in all the three *P. striiformis* pathotypes (31, K, and 46S 119) to see if they possess *dN*/*dS* ratio >1. Pairwise *dN*/*dS* ratio for a particular gene was calculated by considering the mean of *dN*/*dS* ratios of that particular gene with other genes in the same cluster. In total, 10 genes with a predicted signal peptide were under strong positive selection pressure with *dN*/*dS* ratio >1. Four genes each from pathotypes 31 and 46S 119 and two genes from pathotype K had *dN*/*dS* ratios in the range of 1.2 to 1.76 (Tables S21 and S22). All the genes identified could not be annotated (i.e. did not produce significant hits in BLAST search or were hypothetical genes). This could be attributed to the feature that *Puccinia* spp. in general, are among some of those poorly annotated genomes with very less and precise functional categorization of genes even in the best known public databases.

### Predominance of purifying selection in ES proteins of pathotype 46S 119

There are several reports on genes that are evolutionarily conserved and have evolved solely under purifying selection^66^. The investigation of the type of diversifying selection process undergoing on the majority of the ES genes of *P. striiformis* in this study revealed that genes with *dN*/*dS* ratio below unity were highest in pathotype 46S 119 (89) compared to pathotypes 31 (53) and K (35), (Table 23). Because the number of genes analyzed for diversifying selection by CODEML was similar in all the pathotypes: 278 in the case of pathotypes 31 and 46S 119 and 279 in pathotype K, thus indicating that the genes under purifying selection were far more in pathotype 46S 119 compared to the other two pathotypes. Most of the genes in pathotypes 31 (240), K (304), and 46S 119 (236) analyzed for the diversifying selection showed *dN*/*dS* ratios <0 (Table S23). A detailed analysis of the data revealed that, for genes where individual *dN* and/or *dS* values were not detected, all were assigned negative values (<0) by YN00 and CODEML, which otherwise is either equal to zero or infinity. We assigned all such genes with values “0” (Table S24). These results were very contrasting yet interesting and could be explained as follows. These genes either were absolutely identical with no detected nonsynonymous (*dN*) and synonymous (*dS*) mutations or possessed exclusively only any one of the mutations within them. Nevertheless, the results clearly indicated that ES proteins among the three species are well conserved all through, and second, purifying selection has an important role in differentiating pathotype 46S 119 from pathotypes K and 31.

### Evolutionary dissection of *P. striiformis* genomes

**Figure 6:**
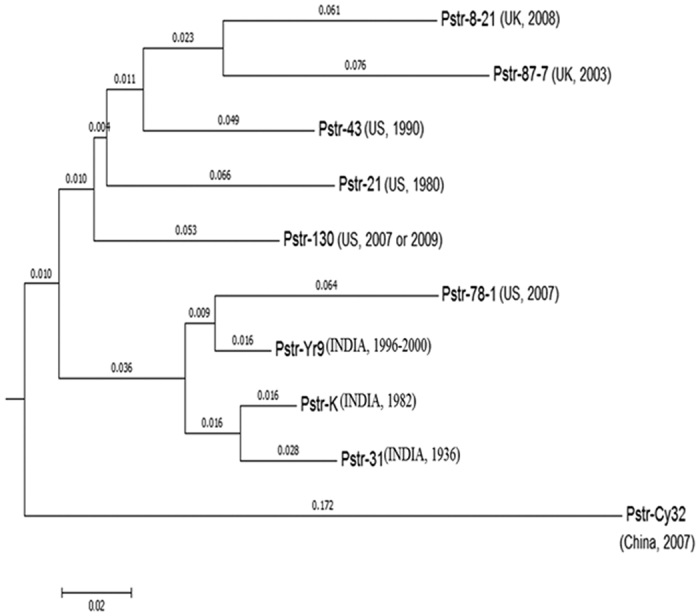
Evolutionary analysis of *P. striiformis* genomes. Neighbor joining tree depicting the evolutionary pattern of ten published *P. striiformis* genome based on genome alignments and conservation distance.

A comparative evolutionary analysis of the 10 genomes of *P. striiformis* pathotypes, which included three pathotypes of this study and seven other published genomes (Tables S2 and S25), resulted in three major clusters. Cluster 1 included all the pathotypes from the United Kingdom and United States, cluster 2 contained all the Indian pathotypes and one U.S. pathotype (78-1), and cluster 3 included the lone pathotype of Chinese (Cy32) origin (Fig. 6a). These results could clearly demonstrate the distribution of pathotypes based on their geographical regions, barring one exception. Furthermore, within the Indian pathotypes, the decreasing conservation distance on each of the branch correlated to their year of detection, indicating pathotype 31 to be the oldest (detected in 1936) among the three.

## Discussion

Yellow rust of wheat caused by *Puccinia striiformis* is a disease of worldwide significance because it results in extensive yield losses every year. This pathogen is highly variable resulting breakdown of resistance of different *Yr* genes. To understand the dynamic nature of the genome of this fungal pathogen, whole genome sequence analysis of highly and least variable pathotypes of *P. striiformis* is of great significance. In this study whole genomes of three pathotypes of *P. striiformis* (31, K, and 46S 119) have been decoded by using next-generation sequencing (NGS) technology. The information on the genome sequence of wheat rust pathogens though already are available from USA, China and UK, but the precise information about genes responsible for pathogenicity is still limited due to the poor functional annotation of predicted genes. Genome-wide identification of genes rich in cysteine residues, pathogen-host interaction-related genes, and ES genes in the three individual Indian pathotypes have generated a huge resource for further validation experiments. Also, categorization of 81% of the total annotated genes into 22 different functional classes in these pathotypes has generated a gene repertoire to perform comparative analyses among various rust fungi. It will also help to understand the essential metabolic pathways undergoing in rust spores during early stages of infection and understanding genetic variability in this important pathogen. Largely, spontaneous mutations have been considered as the major source of genetic variation responsible for the emergence of new virulent pathotypes in the pathogens^67^,^68^. Consistent with this observation, pathotype 46S 119 detected in the year 1996, which probably has emerged through a single-step mutation in pathotype 46S 103 on *Yr9*, and another pathotype 78S84 in the year 2000 from the same place in India have made the exploration of its genome vital to the scientific community^1^. Besides, pathotype 46S 119 is the most virulent pathotype until 2004^69^. The other phenotypic and phylogenetic features of the three *P. striiformis* pathotypes (31, K, and 46S 119) included in this study, wherein pathotype 31 is least virulent among the three pathotypes and avirulent to *YrA, Yr3, Yr4*, and *Yr*9 genes, whereas pathotypes K and 46S 119 are virulent to all these genes, except that pathotype K is avirulent to *Yr9*, made this comparison significant to understand the undergoing mechanism of divergence and evolution pattern among these three pathotypes.

An NGS-based genome assembly, especially with short reads, has always been difficult, giving unsatisfactory results due to the extremely heterozygous nature of the *P. striiformis* genome^12^. The resequencing data generated purely by NGS in the current study for three pathotypes, however, showed an improved assembly compared to the previously published genome of *P. striiformis* pathotype 130^11^. Alternative strategies involving Sanger sequencing have shown better results, for example, in *P. striiformis* pathotype CY32 in China^12^. Although we did not use any such alternate sequencing strategy, still the assembled genome sizes achieved (~66 to ~70 Mb) in the present study were in concordance with the previously assembled genome of the *P. striiformis* pathotype 78-1 from the United States, which was also used as a reference sequence for the genome assembly of three individual *P. striiformis* pathotypes, namely, 46S 119, 31, and K in our study^70^.

Previously, Zheng *et al*.^12^ have revealed that the phylogenetic relationship based on cSNPs of *P. striiformis* isolates could not correlate with their virulence spectrum on differential cultivars or their geographical origins. Similarly, the findings of the present study, which include the phylogenetic analysis performed on whole-genome sequences of three Indian pathotypes along with two pathotypes from the United Kingdom, four from the United States, and one Chinese pathotype, are partially inconsistent with the previous results. The present analysis clearly revealed three different clusters showing a close relationship between pathotypes collected from the same geographical regions, with an exception of the US pathotype (78-1), which showed a close relationship with the Indian pathotypes. In United States, the resistant *Yr8* and *Yr9* genes were effective against all the races identified before 2000. An important group of new races virulent to these genes included *P. striiformis* race-77, 78, 79, and race-80 and were all detected post-2000^71^. Furthermore, an efficient description of the relationship of *P. striiformis* races identified in the United States either before or after 2000 was given by Chen *et al*.^72^. *Yr9* virulence had occurred in Africa, Asia, Europe, and South America^73^ even before its detection in the United States in 2000. Therefore, the appearance of the post-2000 group of *P. striiformis* races in the United States, including race-78 in 2000, was more likely an introduction from outside of the country^1^,^71^,^74^. These studies have raised possibilities of the identification of the Indian pathotype 46S 119 in 2000 and its virulence on *Yr9* to be introduced from outside or as an in-house originated pathotype and/or both the pathotypes shared a common origin. Nevertheless, the US pathotype 78-1 showing a close relationship with the Indian pathotype 46S 119 has supported our evolutionary analyses in being clustered together. Also, in cluster 1, the pathotype from the United States^43^ showed a close relationship with the UK pathotype (8/21). An explanation to this was reported by Cantu *et al*.^75^ in their phylogenetic analysis using the homokaryotic SNP data in both the coding and noncoding regions as well as based on the number of absent genes in pairwise comparisons between US pathotype 43 and UK pathotypes 08/21 and -87/7, which clustered together. Zheng *et al*.^12^ have also suggested that variations associated with pathotype evolution and virulence are the factors responsible for the separate grouping of pathotype CY32 from other Chinese pathotypes. On the contrary, our findings, irrespective of any virulence-based factor, even whole-genome based analysis, showed that race CY32 is grouping away from all other pathotypes. Therefore, the present findings suggested that multifactorial reasons, including virulence, genetic recombination, geographical origins, and mutational events, might be playing an important role during the course of evolution.

Repetitive elements generally are the major components of most of the eukaryotic genomes. Dissecting the genomes of three pathotypes based on the analysis of mobile repetitive elements revealed that more than 36% of all the three genomes is repetitive in nature, with majority being contributed by LTR retrotransposons (>60%). The ratio of content of repetitive elements to genome size is reduced with the increasing genome size in terms of million basepairs among the pathotypes used in the present study, whereas the percentage of intact LTR elements (putative active elements) in pathotype 46S 119 was 5% more than 31 and 2% more than K, indicating that insertion events are active in the three pathotypes. Unlike the two *Fusarium* species, *Fusarium oxysporum* and *Fusarium graminearum*, which differ substantially both in terms of mobile elements and their activity as well as their genome structures^76^, our study did not find such significant difference that could be explained based on repetitive elements to be the factor responsible for the size of *P. striiformis* genomes published so far, which vary between ~64 and 79 Mb with an exception of 110 Mb in the case of *P. striiformis* pathotype CY32.

All successful pathogen infections are subjected to continuous pressure to diversify their mechanisms to overcome the host defense system and ensure nutrient availability while at the same time evade recognition by the host surveillance system. In filamentous plant pathogens, diversifying selection has been shown to act particularly on genes that encode effector proteins^18^,^77^. Effector proteins are ES fungal pathogen proteins and some of these identified proteins are known to activate effector-triggered immunity (ETI)^78^. Of the total extracellular proteins identified in the present study, the majority of the proteins that showed match with the proteins present in the nr-database were hypothetical; furthermore, a major section of the total secretory proteins belongs to the unannotated class. These proteins with unknown functions could possibly include key effector proteins. Investigation on these proteins for the presence of functional domains and features such as high cysteine content and internal mutational events additionally provided sequence-based annotation for the majority of the predicted secreted proteins of unknown function within the three pathotypes. Diversifying selection analysis performed on these proteins identified four proteins each from pathotypes31 and 46S 119 and two proteins from pathotypeK to be under positive selection, but the majority of the proteins were under purifying selection. The number of proteins subjected to purifying selection in pathotype46S 119 (89) were much higher than such proteins in pathotypes31 (53) and K (35), leading to two possible inferences: (i) the most essential and vital ES proteins probably remained conserved in pathotype 46S 119 to maintain their virulence ability as seen in the majority of the pathogens throughout their evolution^79^,^80^ and (ii) genes of pathotype46S 119 presumably are under strong purifying selection compared to the other two pathotypes and are likely to maintain genes that show direct functional relation with the fitness. Therefore, diversifying selection within ES proteins in pathotype46S 119 perhaps pointed towards purifying selection rather than positive selection, and nonsynonymous changes within very few ES genes could be a feature enough for its diverse nature. It has been reported that whole-genome nonsynonymous and synonymous divergence ratio between closely related species or species from the same lineage tends to show *dN*/*dS* < 1 or unity^81^,^82^ and that *dN*/*dS* < 1 can occur under both negative and positive selections. Insuch cases, the relationship between selection and *dN*/*dS* is not necessarily a simple repetitive function, and it may be impossible to infer the selection pressure from the *dN*/*dS* measurement^83^. Therefore, in our study, the majority of ES proteins from pathotype 46S 119 and, to a greater extent, those from pathotypes 31 and K, possessing *dN*/*dS* < 1, might actually be under a strong positive selection because of the deployment of *Yr* genes in all the wheat genotypes being grown in different agro ecological regions.

Self-BLAST of the assembled contig sequences of the three *P. striiformis* genomes against each other enabled the prediction of duplicated genomic regions. The estimation of overrepresented regions of three genomes by percentage-based alignment identity revealed that pathotype 46S 119 has a higher level of SD than the other two pathotypes. This could be explained as pathotype 46S 119 is one of the most recent, virulent, and prevalent pathotypes, although the difference in genome SDs did not correlate to the percentage of ES diversifying genes (~6.47) in any of the three genomes. Furthermore, functional annotation of the genes under duplication supported the fact that these duplicated regions might be playing an important role in the recruitment of genes related to energy metabolism, mobile and extra chromosomal element, and protein metabolism with very few genes of cellular process, which generally contain all secreted proteins. A similar study by Wong *et al*.^84^ showed that gene duplications are only partially responsible for the adaptation of platypus venom (a secreted protein); thus, the major cause of differences among the three pathotypes, especially for genes directly involved in or during the establishment of the pathogen on the host and/or in early infection and disease establishment, probably is due to mutational events (SNPs, insertions, and deletions). On similar lines, our SNP analysis also revealed that pathotype 46S 119 is potentially more variable than the much older pathotypes K and 31. The above analysisalso indicate that gene conversion or neofunctionalization of genes might have little role to play in the variability of these pathotypes.

The genus *Puccinia* is a biotrophic pathogen contains many species infecting a large number of plant species. Although genome sequence of some *P. striiformis* pathotypes are available in the public domain, still precise annotation of the genes could not be done because of unavailability of precise functional gene validation tools. However, extensive information on the complete genome sequences of three pathotypes of *P. striiformis* and annotation of repetitive elements and genes generated in this study will help in further understanding of variable nature of this important wheat pathogen. The present study has greatly helped in understanding the three *P. striiformis* genomes and their structural features and identified genes coding for secreted proteins that can be considered to be a vital source for extending studies in thispathogen which will further help in designing strategies to managestripe rust disease in wheat.

## Additional Information

**Accession codes:** Assemblies were deposited in the NCBIGenBank and their BioProject IDs and WGS accession numbers are listed as follows:

***PucciniastriiformisPathoype*:** 31(67S64); K(47S102); 46S1119(Yr9 vir.)

**BioProject ID:** PRJNA277552; PRJNA277553; PRJNA277554

**Accession numbers:** LASC00000000; LACT00000000; LACU00000000

**How to cite this article:** Kiran, K. *et al*. Dissection of genomic features and variations of three pathotypes of *Puccinia striiformis* through whole genome sequencing. *Sci. Rep.* **7**, 42419; doi: 10.1038/srep42419 (2017).

**Publisher's note:** Springer Nature remains neutral with regard to jurisdictional claims in published maps and institutional affiliations.

## Supplementary Material

Supplementary Information

Supplementary Dataset 1

## Figures and Tables

**Table 1:** Description of the pathotypes of *Puccinia striiformis* used for sequencing.

S. No.	Old Name	New Name	Year of Detection	Place	Isolated from	Susceptible Yr genes/Lines
1	31	67 S 64	1936	Shimla	Local cultivar	*Yr*2
2	K	47 S 102	1982	Punjab	—	Sonalika
3	*Yr9* virulence	46S 119	1996	Gurdaspur	CPAN3004	Sonalika, Kalyansona, *Yr*9

**Table 2:** Assembly and gene prediction statistics of the genomes of three pathotypes of *P. striiformis*.

Data Type	*P. str*31	*P. str*K	*P. str*46S 119
Input reads (No)	70,840,540 (6.97 Gb)	87,617,660 (8.59 Gb)	62,525,768 (6.11 Gb)
Total contigs (Assembled genome)	30,066 (66.26 Mb)	32,818 (69.77 Mb)	24,737 (70.24 Mb)
N50 (contigs) (bp)	7434	8463	9257
Average contig length (bp)	2203	2126	2839
GC content of assembled genome (%)	44.43	44.41	44.40
Largest contig (bp)	54818	67807	73102
Contigs ≥2 K	7891 (54.77 Mb)	7470 (56.84 Mb)	7703 (59.91 Mb)
Contigs ≥200 bases	22212 (65.18 Mb)	24642 (68.65 Mb)	20261 (69.59 Mb)
Average contig length (>2 k)(bp)	6941	7609	7778
N50 (>2 k contigs) (bp)	9536	11060	11597
Depth of coverage	105X	123X	86X
Repeats	24.39 Mb	25.31 Mb	24.75 Mb
Repeats % in assembled genome	36.80	36.27	35.23
Number of genes predicted	18362	18880	19795
Mean gene length (bp)	1070	1075	1072
Total Number of Exons	86407	89695	93608
Mean number of exons per gene	4.70	4.75	4.72
Largest Gene Length (bp)	13,479	13,494	16,128
Genes (> = 150 bases)	17,103	17,569	18,375
Genes (> = 450 bases)	12,354	12,669	13,216
Average gene length (bp) (> = 450 bases genes)	1467	1479	1482
Mean number of exons per gene (> = 450 bases genes)	5.68	5.75	5.74
