# Supplementary material for: Dissection of genomic features and variations of three pathotypes of Puccinia striiformis through whole genome sequencing
Source: Sci Rep. 2017 Feb 17;7:42419. doi: 10.1038/srep42419 (PMC5314344; doi:10.1038/srep42419)
Supplement: Supplementary Information [file srep42419-s1.pdf]

Dissection of genomic features and variations of three pathotypes of *Puccinia striiformis* through whole genome sequencing

Kanti Kiran[1], Hukam C Rawal[1], Himanshu Dubey[1], Jaswal R[1], Subhash C Bhardwaj[2], P Prasad[2], Dharam Pal[3], Devanna B N[1],Tilak R Sharma[*1]

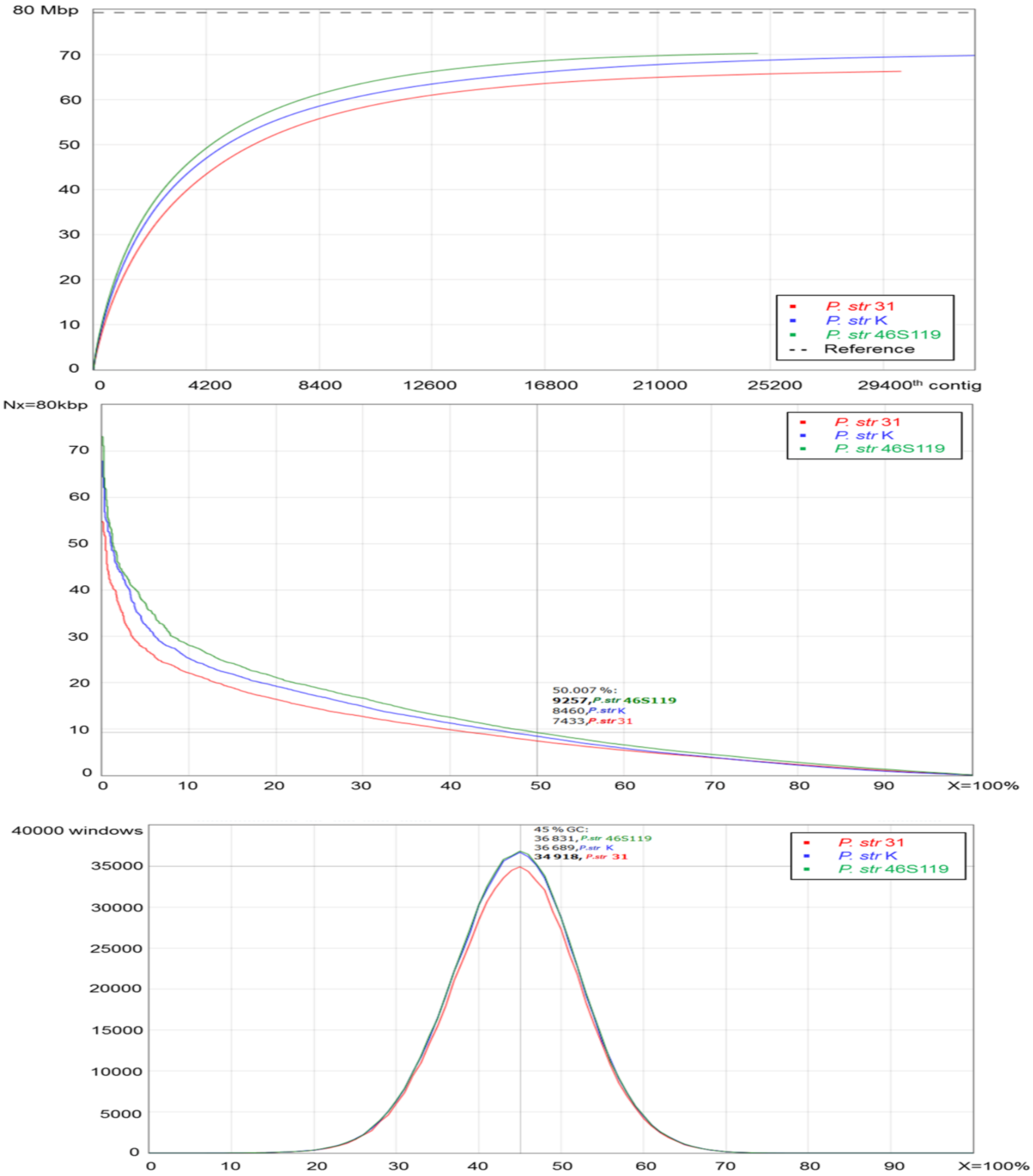

Supplementary Figure 1 Assembly statistics of P.striiformis pathotypes 31, K and 46S119 by QUAST 3.2. Cumulative length of contig versus the genome size in all the three pathotypes (31, K and 46S119), post assembly, contigs are ordered from largest (contig #1) to smallest (**extreme top figure**). Presentation of N50 as a quality of assembly in the three pathotypes (**middle figure**). Whole genome presentations of GC% in the pathotypes, contigs are broken into nonoverlapping 100 bp windows. Plot shows numbers of windows for each GC percentage (**bottom figure**). Reference used is from Broad Institute *P. striiformis* isolate 2K41-*Yr9*, Race 78.

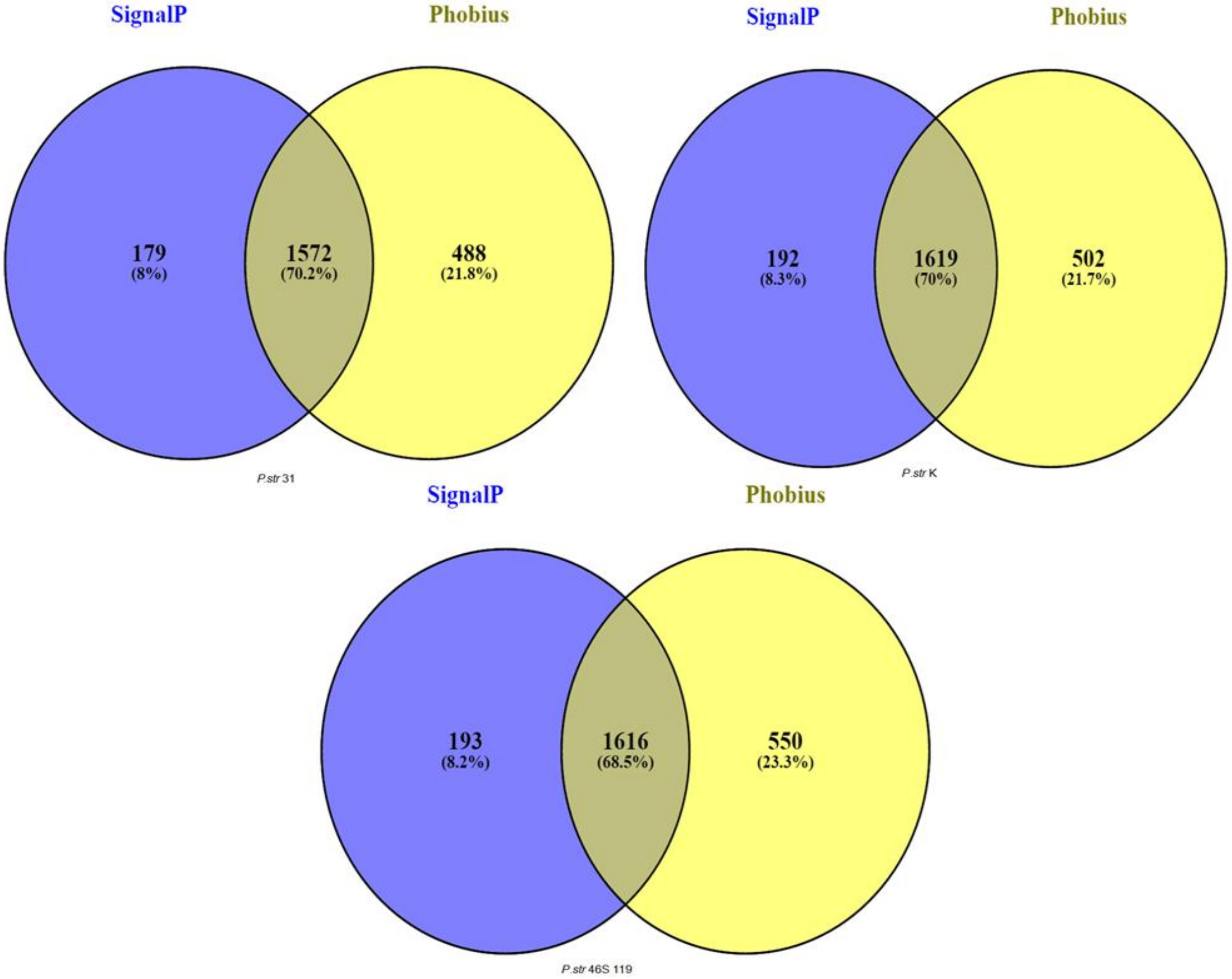

Supplementary Figure 2. Venn diagram representation of secretory proteins predicted with signalP and Phobius .

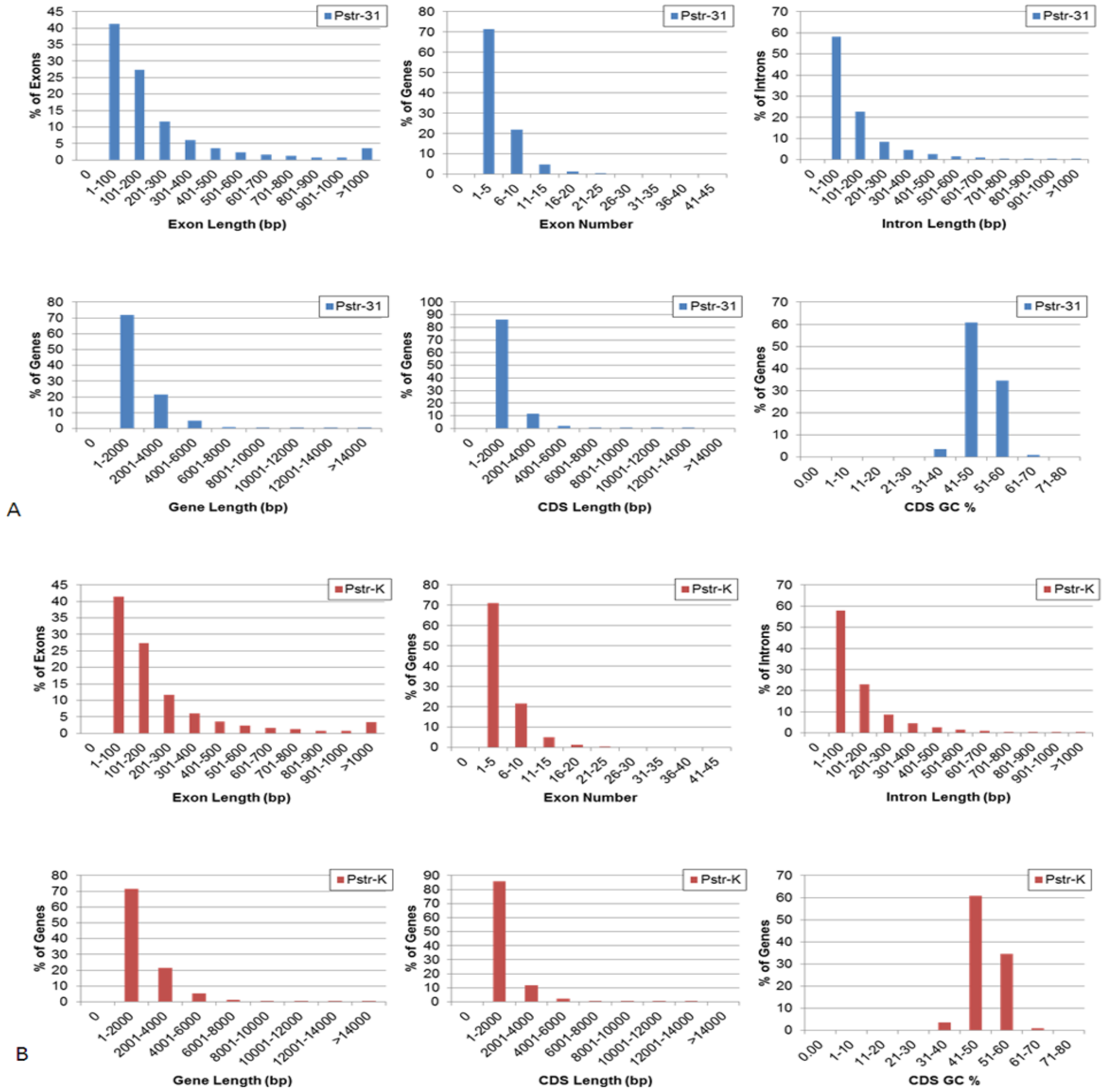

Supplementary Figure 3a. Gene prediction and annotation in the genomes of three pathotypes of *P.* *striiformis*. Analysis of the genome sequence of pathotypes 31 and K .

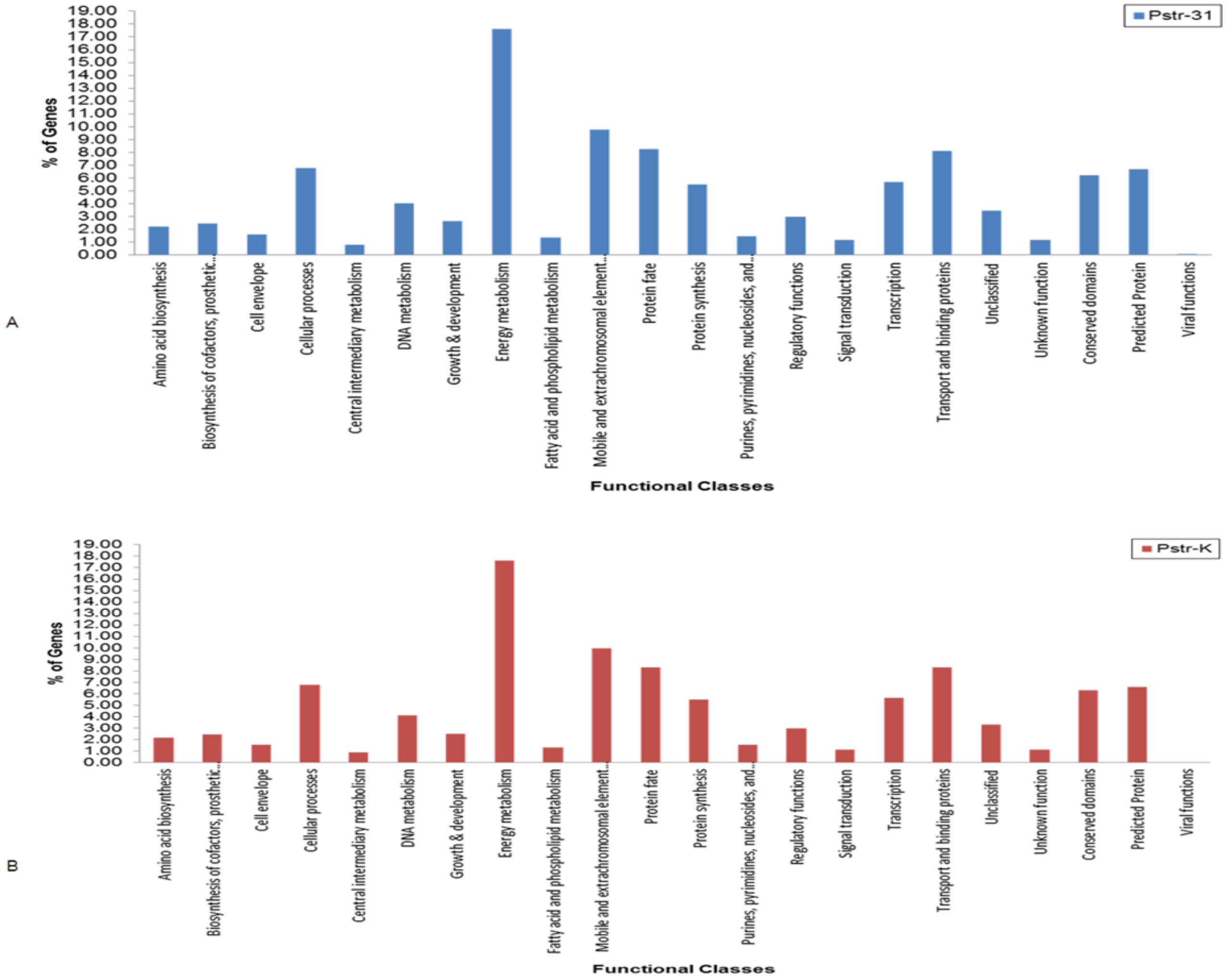

Supplementary Figure 3b. Functional annotation of genes of pathotypes P.str 31 and P.str K.

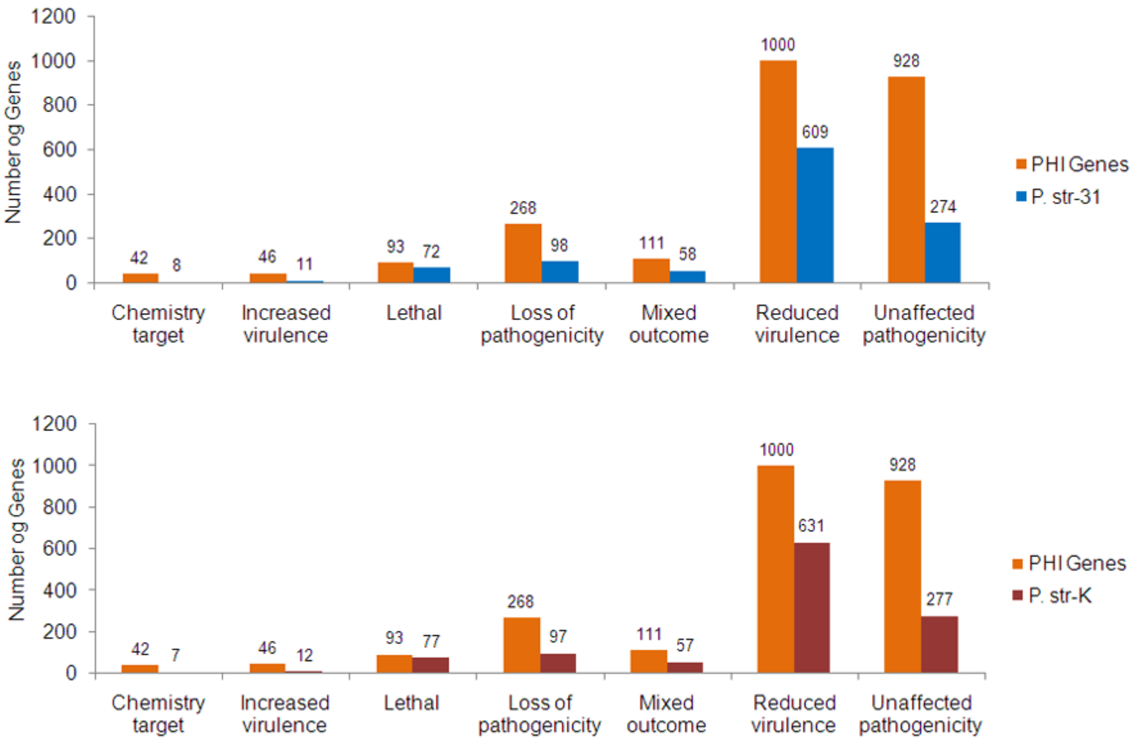

Supplementary Figure 4. Annotation of the genes of pathotypes P.str 31 and P.str K analysed by PHIdb.

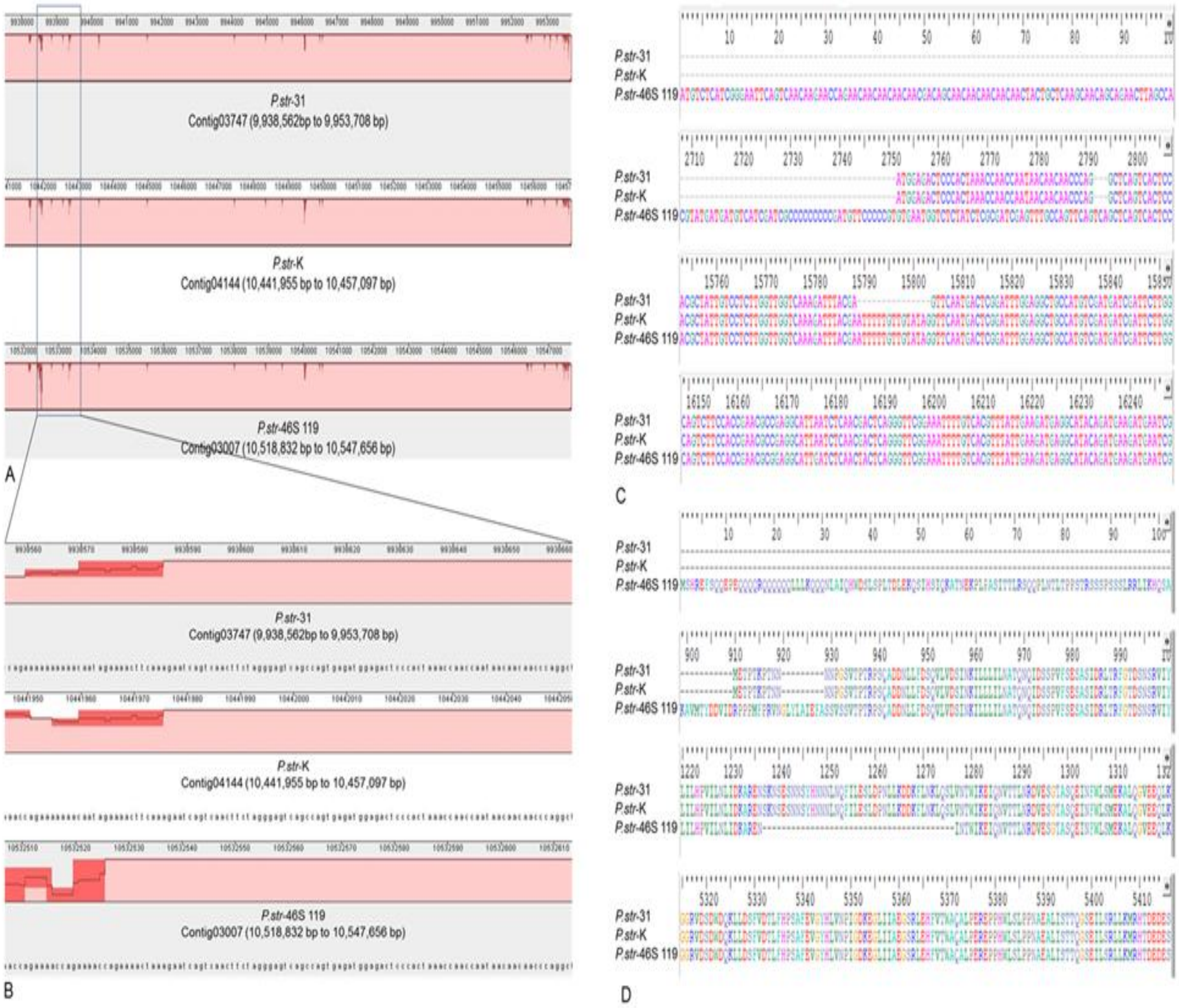

Supplementary Figure 5. Contig specific analysis of site specific recombination event in the three pathotypes viz. P.str 31, P.str K and P.str 46S 119.

Supplementary Table S1 Statistical analysis of the genome assembly of pathotypes 31 K and 46S119. Report generated by QUAST 3.2 tool. Ref. (Pt. 2K41-Yr9, Race 78) size: 79310008 bp , G+C content: 44.43 %

**Statistics**	***P. str* 31**	***P.str* K**	***P.str* 46S 119**
# contigs	30066	32818	24737
# contigs (>= 5000 bp)	3774	3698	3809
# contigs (>= 10000 bp)	1606	1767	1809
# contigs (>= 25000 bp)	132	213	284
# contigs (>= 50000 bp)	5	13	15
Largest contig	54818	67807	73102
Total length	66263768	69776778	70246360
Total length (>= 5000 bp)	41431250	44646267	47360216
Total length (>= 10000 bp)	26181090	30975563	33285837
Total length (>= 25000 bp)	4255806	7138586	9546470
Total length (>= 50000 bp)	265453	730400	877850
N50	7434	8463	9257
N75	3083	3032	3656
L50	2410	2192	2000
L75	5858	5647	5045
GC (%)	44.44	44.41	44.41
Misassemblies			
# misassemblies	42	30	13
# relocations	0	0	0
# translocations	42	30	13
# inversions	0	0	0
# misassembled contigs	39	27	13
Misassembled contigs length	12651	11595	4288
Mismatches			
# mismatches	360071	387638	196449
# indels	36320	41977	20223
Indels length	75614	86260	36401
# mismatches per 100 kbp	552.16	560.58	280.72
# indels per 100 kbp	55.7	60.7	28.9
# short indels	34388	39859	19445
# long indels	1932	2118	778
Genome statistics			
Genome fraction (%)	82.223	87.189	88.235
Duplication ratio	1.001	1	1
Largest alignment	54812	67807	73102
NG50	5465	6620	7463
NG75	1103	1474	2100

Worst Median Best

Supplementary Table S2 . Genomes used for Comparative Phylogenetic study

Organism/Name	Strain	BioSample	BioProject	Size (Mb)	GC%	WGS	Release Date	Modify Date
Puccinia striiformis f. sp. tritici CY32	CY32	SAMN02981480	PRJNA176877	130.485	44.8	ANHQ01	30-09-13	11-08-14
Puccinia striiformis f. sp. tritici PST-130	PST-130	SAMN00025370	PRJNA51241	64.7866	44.5	AEEW01	16-08-11	04-08-14
Puccinia striiformis f. sp. tritici PST43	PST43	SAMN01818050	PRJNA181957	70.6739	44.2	AORQ01	01-03-13	25-04-13
Puccinia striiformis f. sp. tritici PST21	PST21	SAMN01818051	PRJNA181959	73.0475	44.4	AORR01	01-03-13	25-04-13
Puccinia striiformis f. sp. tritici 08/21	08/21	SAMN01818052	PRJNA181960	56.2671	44.2	AORS01	01-03-13	25-04-13
Puccinia striiformis f. sp. tritici 87/7	87/7	SAMN01818053	PRJNA181962	53.3988	44.1	AORT01	01-03-13	25-04-13

6 “Genome Assembly and Annotation report” entries were found on the date for Puccinia striiformis (or ID: 2580) while looking for available genomic data in NCBI. We downloaded the genomic data and compared them with our three pathotypes and 1 reference genome to find out the phylogenetic relation between them, if any.

Publications

1. Genome analyses of the wheat yellow (stripe) rust pathogen Puccinia striiformis f. sp. tritici reveal polymorphic and haustorial expressed secreted proteins as candidate effectors. Cantu D, et al. BMC Genomics 2013 Apr 22.

2. Next generation sequencing provides rapid access to the genome of Puccinia striiformis f. sp. tritici, the causal agent of wheat stripe rust. Cantu D, et al. PLoS One 2011.

3. High genome heterozygosity and endemic genetic recombination in the wheat stripe rust fungus. Zheng W, et al. Nature Communications, 2013.

Supplementary Table S3. Reference mapping against *P. striiformis* pathotype 78-1 and back reference assembly

Pathotype	*P.str* 31	*P.str* K	*P.str* 46S 119
Raw Reads	70,840,540 (6.97 Gb)	87,617,660 (8.59 Gb)	62,525,768 (6.11 Gb)
Mapped Reads	55,939,570 (5.48 Gb) (78.96%)	64,841,092 (6.31 Gb) (74.00%)	44,068,854 (4.32 Gb) (70.48%)
Assembled Genome	30,066 (66.26 Mb)	32,818 (69.77 Mb)	24,737 (70.24 Mb)
De-novo Assembly of unmapped reads	133,379 contigs (22.80 Mb)	155,208 contigs (33.71 Mb)	381,332 contigs (142.94 Mb)
Back Reference Assembly (Reads Mapped)	55,306,839 (5.40 Gb) (78.07%)	64,384,858 (6.23 Gb) (73.48%)	43,397,120 (4.23 Gb) (69.41%)
Back Reference Assembly (Assembled Genome)	29,785 contigs (66.16 Mb)	32,811 contigs (69.65 Mb)	24,666 contigs (70.19 Mb)

Supplementary Table S4. Intra-pathotype SNPs to evaluate diversity within the genomes

Mapped Reads	Reference	Total SNPs	SNPs/Kb
Pstr-31	*Pstr*-31	454128	6.85333801
Pstr-K	*Pstr*-K	146802	2.10388046
Pstr-46S 119	*Pstr*-46S 119	489617	6.96999816

Supplementary Table S5. Inter-pathotype SNPs to evaluate the diversity across the genomes

Mapped Reads	Reference	Total SNPs	Heterokaryotic SNPs/Kb	Homokaryotic SNPs/Kb
Pstr-31	*Pstr*-K	5,98,272	4.26	0.67
Pstr-31	*Pstr*-46S 119	7,85,625	4.23	3.45
Pstr-K	*Pstr*-31	5,42,867	3.58	0.71
Pstr-K	*Pstr*-46S 119	7,49,756	3.66	3.48
Pstr-46S 119	*Pstr*-31	9,67,260	6.22	1.467
Pstr-46S 119	*Pstr*-K	9,54,696	6.04	1.64

Supplementary Table S6 Total annotated genes in the three *P. striiformis* pathotypes

Description	***P.str* 31**	***P.str* K**	***P.str* 46S 119**
Total predicted Genes	18362	18880	19795
Genes (>=450 bp)	12354	12669	13216
Annotated genes	10082 (81.6%)	10328 (81.5%)	10774 (81.5%)
Annotated genes (excluding Hypothetical)	5795	5921	6103

Supplementary Table 7 Functional annotation of the genes predicted in *P. striiformis* pathotypes

S.No	Functional Class	*P.str* 31	*P.str* K	*P.str* 46S 119
1	Amino acid biosynthesis	2.23	2.14	2.24
2	Biosynthesis of cofactors, prosthetic groups, and carriers	2.45	2.47	2.39
3	Cell envelope	1.59	1.54	1.57
4	Cellular processes	6.78	6.79	6.60
5	Central intermediary metabolism	0.81	0.88	0.90
6	DNA metabolism	4.00	4.12	3.98
7	Growth & development	2.64	2.48	2.46
8	Energy metabolism	17.64	17.60	17.42
9	Fatty acid and phospholipid metabolism	1.38	1.33	1.23
10	Mobile and extrachromosomal element functions	9.77	9.96	10.93
11	Protein fate	8.23	8.28	8.19
12	Protein synthesis	5.50	5.51	5.44
13	Purines, pyrimidines, nucleosides, and nucleotides	1.47	1.54	1.43
14	Regulatory functions	2.97	2.99	2.92
15	Signal transduction	1.16	1.10	1.11
16	Transcription	5.69	5.62	5.55

17	Transport and binding proteins	8.11	8.29	7.91
18	Unclassified	3.47	3.33	3.51
19	Unknown function	1.19	1.11	1.26
20	Conserved domains	6.21	6.32	6.31
21	Predicted Protein	6.66	6.57	6.60
22	Viral functions	0.05	0.03	0.03

Supplementary Table S8 .BLAST search of predicted genes (from unmapped reads) against *P.striiformis*78-1

*Significant hits = BLAST hits with E-value ≤ e-10 &Bit score ≥ 100

**Pathotypes**	**Genes predicted**	**Significant Hits with reference***	**Hits with 100% Identity**	**Genes with no significant hits**	**% of genes with no significant hits**
P.str-31	11333	251	0	11082	97.78
P.str-K	27872	508	7	27364	98.17
P.str-46S 119	97011	1868	9	95143	98.07

Pathotypes	Genes predicted	Significant Hits with reference*	Hits with 100% Identity	Genes with no significant hits	% of genes with no significant hits
P.str-31	11333	251	0	11082	97.78
P.str-K	27872	508	7	27364	98.17
P.str-46S 119	97011	1868	9	95143	98.07

Supplementary Table S9. Inter-species BLAST search of predicted genes from unmapped regions between three pathotypes

Query	Genes with 100% identity and same length			Genes with no significant hits in any of the other 2 pathotypes
	***P.str*-31**	***P.str*-K**	***P.str*-46S 119**	
P.str-31	-	128	62	10,050 (88.67%)
P.str-K	128	-	66	24,969 (89.58%)
P.str-46S 119	62	66	-	94,183 (97.08%)

Supplementary Table S10: Number of predicted PHI related genes and their percentages in *P.striiformis* genomes

**PHI based Functional Class**	***P. str*-31**	***P. str*-K**	***P. str*-46S 119**
Chemistry target	8	7	7
Increased virulence	11	12	11
Lethal	72	77	75
Loss of pathogenicity	98	97	101
Mixed outcome	58	57	56
Reduced virulence	609	631	641
Unaffected pathogenicity	274	277	274
Total	1130	1158	1165
**Percentage of predicted PHI related genes**			
**Function**	***P. str*-31**	***P. str*-K**	***P. str*-46S 119**
Chemistry target	0.71	0.60	0.60
Increased virulence	0.97	1.04	0.94
Lethal	6.37	6.65	6.44
Loss of pathogenicity	8.67	8.38	8.67
Mixed outcome	5.13	4.92	4.81
Reduced virulence	53.89	54.49	55.02
Unaffected pathogenicity	24.25	23.92	23.52

Supplementary Table S11 Correlation of different outcomes with the year of detection/isolation of *P. striiformis* pathotypes

**Description**	***P.str* 31**	***P.str* K**	***P.str* 46S 119**
Year of detection	1936	1982	1996-2000
Genome Size (Mb)	66.26	69.77	70.24
Largest Contig (Kb)	54	67	73
Genes Predicted	18362	18880	19795
Genes Annotated against NR	10082	10328	10774
Genes Annotated against PHI	1130	1158	1165
Tandem Repeats Numbers	14,371	15,067	15,285
Tandem Repeats(Kb)	788	832	851
SSRs	14,725	15,335	15,353
SDs length (Mb)	1.43	1.61	2.30
SDs % in Genome	2.15	2.30	2.89
Tandem Repeats Length % in genome	1.18	1.19	1.21
Genes Annotated against NR %	54.90	54.70	54.42
Genes Annotated against PHI %	6.15	6.13	5.89
Repeats (TE) Length % in genome	36.80	36.27	35.23

Supplementary TableS12 Identification of full length LTR elements in *P. striiformis* genomes

Genomes	Total LTRs	LTR+TSR	LTR-TSR
P.*str* 31	43	20	22
P.*str* K	46	21	25
P.*str* 46S 119	57	27	30

Supplementary TableS13 Identification of secretory proteins in *P. striiformis* pathotypes

Stage A analysis (A)					
			**P.** ***str*** **31**	**P.** ***str*** **K**	**P.** ***str*** **46S 119**
Level I	**Total protein >30 a.a**		17280	17750	18561
	**SignalP 4.1**		1751	1811	1809
	**TargetP 1.1**		2880	2986	3071
Level II	**Unique secretory proteins**		3924	3061	3123
	**TmHmm**				
		**0TM**	2215	2309	2358
	<10 a.a of TM in mature peptide	**1TM3**	377	382	412
Level III	**Total secretory proteins**		2592	2691	2770
	**ProtComp 10**		704	747	741
	**Mature protein length < 20aa**		17	20	21
Level IV	**Secretome**		687	727	720

Supplementary TableS14 Identification of sub cellularly localized proteins in *P. Striiformis* pathotypes

Stage B analysis			
	**P. *str* 31**	**P.*str* 46S 119**	**P.*str* K**
**WolfPsort 3**			
> 17 a.a	248	256	261
≤ 17 a.a	238	252	243
Other	201	212	223
Filter sequences with			
**GPI**	6	9	5
**1TM**	29	44	39
**Starting a.a not (M)**	2	3	4
**Refined secretome**	211	200	213

Supplementary TableS15 Secretory proteins with precise function specific to the three *P. striiformis* pathotypes

**Proteins**	***P. str* 31**	***P. str* K**	***P. str* 46S 119**
adenylyl cyclase-associated protein 1	1	1	1
alpha-galactosidase	0	0	1
endonuclease	1	1	1
glyceraldehyde-3-phosphate dehydrogenase	0	0	1
glycosyltransferase family 39 protein	1	1	1
hAT family dimerization domain-containing protein	0	0	1
secreted protein	1	1	1
sterol 24-C-methyltransferase	0	0	1
ubiquitin-conjugating enzyme E2 J2	1	1	1
copper/zinc superoxide dismutase	1	2	2
differentiation-related protein 1	1	0	0
plasma membrane proteolipid 3	1	0	0
ubiquitin-activating enzyme E1	0	1	0

Supplementary TableS16 Clusters of Orthologous genes present in the three *P.str* genomes

Genus	Genomes	Genes in clusters
1	P. *str* 46S119	1
2	P. *str* 31 and P. *str* 46S119	34
	P. *str* 31 and P. *str* K	74
	P. *str* K and P. *str* 46S119	65
3	P. *str* 31, P. *str* 46S119 and P. *str* K	514

Supplementary Table S18 Diversifying selection analysis for the genomes of three pathotypes of *P. striiformis* across extracellular secreted proteins (≥100aa; starting with M).

	***P. str* 31**	***P. str* K**	***P. str* 46S119.**
No.ofGenes analyzed (YN00/CODEML)	347/278	341/279	329/278
Mean *dN/dS*ratio	0.08	0.05	0.12
% of genes with sites under diversifying selection	6.47	6.45	6.47

Supplementary Table S19 . Extracellular secreted genes under site specific diversifying selection within the *P. striiformis* pathotypes

s.no.	ID	Annotated_Function	Length (aa)
1	PSTR_31_00039_2	Not annotated	245
2	PSTR_31_01082_2	Not annotated	108
3	PSTR_31_06674_3	Not annotated	122
4	PSTR_31_06948_4	Not annotated	252
5	PSTR_31_08968_2	Not annotated	345
6	PSTR_31_09159_1	Not annotated	234
7	PSTR_31_09728_1	hypothetical protein	166
8	PSTR_31_10753_1	Not annotated	160
9	PSTR_31_10916_3	Not annotated	185
10	PSTR_31_13228_3	Not annotated	119
11	PSTR_31_13377_2	Not annotated	137
12	PSTR_31_14047_1	Not annotated	208
13	PSTR_31_14283_4	putative f5 8 type c domain protein	581
14	PSTR_31_14566_2	Not annotated	397
15	PSTR_31_14585_12	Not annotated	211
16	PSTR_31_14974_1	Not annotated	298
17	PSTR_31_15706_1	Not annotated	108
18	PSTR_31_15792_2	hypothetical protein	247
19	PSTR_K_00038_4	Not annotated	270
20	PSTR_K_01210_2	Not annotated	108
21	PSTR_K_07248_3	Not annotated	147
22	PSTR_K_07556_4	Not annotated	252
23	PSTR_K_09855_2	Not annotated	353
24	PSTR_K_10081_1	Not annotated	108
25	PSTR_K_10703_1	hypothetical protein	178
26	PSTR_K_11743_4	Not annotated	160
27	PSTR_K_11954_3	hypothetical protein	295
28	PSTR_K_14315_3	Not annotated	119
29	PSTR_K_14449_2	hypothetical protein	162
30	PSTR_K_15086_1	Not annotated	208
31	PSTR_K_15401_5	putative f5 8 type c domain protein	581
32	PSTR_K_15644_3	Not annotated	457
33	PSTR_K_15672_11	Not annotated	211
34	PSTR_K_16133_2	Not annotated	298
35	PSTR_K_17012_1	Not annotated	108
36	PSTR_K_17117_2	hypothetical protein	266
37	PSTR_Yr9_00029_4	Not annotated	283
38	PSTR_Yr9_00877_2	Not annotated	103
39	PSTR_Yr9_05355_3	Not annotated	147
40	PSTR_Yr9_05564_4	Not annotated	285
41	PSTR_Yr9_07161_2	Not annotated	261
42	PSTR_Yr9_07312_1	Not annotated	139

43	PSTR_Yr9_07780_2	Not annotated	126
44	PSTR_Yr9_08560_4	Not annotated	171
45	PSTR_Yr9_08691_3	Not annotated	185
46	PSTR_Yr9_10469_2	Not annotated	118
47	PSTR_Yr9_10579_3	hypothetical protein	152
48	PSTR_Yr9_11114_1	Not annotated	391
49	PSTR_Yr9_11294_5	putative f5 8 type c domain protein	581
50	PSTR_Yr9_11503_2	Not annotated	437
51	PSTR_Yr9_11517_11	Not annotated	211
52	PSTR_Yr9_11853_1	Not annotated	272
53	PSTR_Yr9_12449_1	Not annotated	167
54	PSTR_Yr9_12515_2	hypothetical protein	247

PSTR_Yr9, is pathotype 46S 119, used as such in the text and analyses else where.

Supplementary Table S20 FastaQ sequences of genes with site specific diversifying selection with significance threshold of P < 0.05 BY CODEML model tests

>PSTR_31_00039_2<br>MPSTHITRAAVIGVFANVIQVSSLPAITPASAAAQPASASSEEMMTSMPTSTNRAAAVAPTTPSTTLSSAEVPMSPPTSSSPASPTATIPKTVSPSGVVPAAAVVENTTAPATIKPSVSTNISSAQPNAASALPVTQAPGTTASVAPTTSPVSTITAPVTPTTAPASAITAPITSTTVPVEAVDTTSRTVTTASKPAIAAPVETFSAGNSSSTANQAARNAVASSSSDSTDASSSANSQSIFWAA
>PSTR_31_01082_2<br>MLQLSGLMACVLLLLVAAPWEATSQEPVKIWFGCNKNVDALCVDKVDKDHRQLTFAERRKPHTRDYACWATLTPYCCWQGKFKLWEDPGWTRRVKTKELDDCKEGGQ
>PSTR_31_06674_3<br>MKLCEMLFLLGVILAEGAFVAANFGCHVDKRFTKGWCALPVTTDPATEKVLTYQMARGAVRSSGAFACPDDSRLVFSCCGATFDVSSTQIIDFNVYGSNCDPHRELMKSRVSQISPESDLN
>PSTR_31_06948_4<br>MNWLQLAILPVVICYFNIMMAATALREEMLNPVLDDHTIFANLDDIPEGNLEPKRRRIAEDHSDVIDSNPDSPNSSADSFDTLMSSSLSGFSDHPHNPGSTVDRESWEMDTPHKTEAHRVPSSWGSGGSTKASIEAQGEMIQALREHFRQSKVLVEAKQGSQRNSGISRSTNERTGFNWPKDPTPLKDEVGFEENQFTTTGERKRPYGSNEGPIAGRENRIDAQKAEIAELYNIGKLKQVERNWRGKQTGG
>PSTR_31_08968_2<br>MHHSFFTTALLIVSNVMAQDPSLSTTGLAPGVAPGVAPGSSGSTTSPTPGTGTGGGAGNGGGSGNGGGVGNGVGNGGGSGNGGGSGNGGGVGNGGGAGNGGNNGNGGAPVTPTPPNGPKPIRVVCANSNLPFSERELAEMAAMDTSSNSTQPTPDPKTCKYTYATPQRHLSSPRDSLPSVSEVQTAAICKNADLTAVCVLNSCNITTAPAPVCQSCYEYTPNPTGDDGIVGTVLTPQVTCEDSYNFNQTDVKTPWVCSDKKQKTFSCGSCAGARACDICYDVKDIPTNPPTTNPPTTNPPTTTPTTPTPSTSPPTPITPSLPATSNTPTTPNPVNPPTTAPATS
>PSTR_31_09159_1<br>MAKLSQLALNCALLIIFLVGTSSSAPDYSSLLTRDVRPDFDCHRTVADCEKRVPANKCSMSSCHTSAQGVEQELQVPAVVKEVVPKSPKASLARDFKTGTHPSFFGSNANNAALPLVYIFHSEYAVCTRHATPDEFKDGKGADLMAKRAAHVRGDPGEVFNCVGESIKTKFANKVAERAYCCGREVNIHGNDDDKLPAELSKVYAIYPEGIYSRLTCEFRDSSLSADPYRNSP
>PSTR_31_09728_1<br>MIMPLSRSGAGFWYPVLMLTIFVFRMVDAHAGIIKPPHLEPGAEFRRQCGQQAFGIVKGAPFISQESWKKRSRQCKDRICQGLFVKDQNLAAVPNFAPGAKIPVSIDVQIPHGGVARLLLVDMKNEKTFQSDGKDIVLQDMVEIVTATLPTATPKECSEPGRCAIS
>PSTR_31_10753_1<br>MNSSSFLTSLSVAFLIQAQILLVQAFLCTDSNVVSLGQTQPVCAIFFDGTHKALADQEGEPPSAPPGSTGQSGGGTGGGTGGGSAGGKGSGTGKGSGTGKGSGTGKGNGGKGESDPFAYHIGKPTVVKDKHNKVIDYLCKPRHQTCCEQPPVLISLWRS
>PSTR_31_10916_3<br>MLGKIQFLLLATLITPQFTFGVLGWTLPHTNTDFPSAVNERRSLIFKNGCNCRRIMVDLKVELTSPFGVKTRKFLLLYTGTVFRPIFDSREEVEMVVEEKAEDGARAAIKDAKSGDAQVNKALGIVGTDGKKLLAGLEKLKDVAANPNSSKEQINSQLAAVNKLREPLLGGCYVVGHKNFKKEH
>PSTR_31_13228_3<br>MHLSSLLQILVVILIQGGVTHVQSWGCEKAGNDYRSAGCIYILPDLNPPSFGQKPRPWHLKLMISPWDNVKKTYSCDKAPQGFKTQACCTSDKVLHDGTTTGIFVGICKKLDGTEFKF

>PSTR_31_13377_2<br>MAVLATPSLLLLLLSPLLPVTAQDKLVDCKYSSPQGASYDFNNLIDPKSWPLNLTTITSTPPSTTIETILVSICKKLPTDHQT<br>QNPPTSCPDRTLVCLLIYNEIAAGLDRRLEQIIPVGLDTLPTPTYIALRQIGNQ
>PSTR_31_14047_1<br>MRVSCPSFPSVPLLSITLWLHPHTCAASLPFPALLNSTSAPYGIMQLADRLLPQPELPYHPVLRIFPRSTPLIPATAPIQPPL<br>PPSSTTFPPSCLPAIAVPRRVSTNPAPLPELPLMPLRNTFDGNTSAYQAAMIVWFDNRRVIQSYLNIPFNQPMPALEESA<br>AIQFSQRAAPATPVIDINDIQTPVGSPITACVKDSPPPMLRSIC
>PSTR_31_14283_4<br>MKLFHFLGSSLLLGSGSAARIGLTAQYFQVDGSDENSIKLSWPADPTVHKFSVIRQSAGTSSSVIAQVQGNIYDDYGVPE<br>GELTYQVLVEGDPSKVSDQVTISSQPSFDSASSLSIYDNTQASNLKAIANIKLESTYYHFNIENDHDGVTQIIETTSTDGYTF<br>SGTKRVLLTRKELCSGSPDGFCKLESASFVQNPKNSEVVMWAHWEKGGPDYGQARVAVAFGQPGGEWEFGGSFRPL<br>ENQSRDLTFFHDNDGSGYLISSTAMNTNLNIYKLTPDWHNVTSLVSTVLKGQRREAPSLINHENVYYLFTSQASDWYPS<br>AGQYISATSLSGPWSQSRNIGNLAGFGAQSGSVQKIGSSWVMCANQWSGQWLDPEPPSHRVILPISLSNGYADYHYY<br>HRLRYDEAGIYGVQDGKVISVGQKCSSASASVPGFEESKSINGVNLDPNNFYLSATVPFSYEIEFEVASILSRFDLTTKLVG<br>GSETAYQFTISGRVSSTSLFRVILNKSNNTRVGFISSKVTDLTTYSAVRLDVHQVLNVHNKKQASWAQGIGQFTVYGHH<br>HQNSPTKSPDLKSARFSKRLLVGR
>PSTR_31_14566_2<br>MFSHKFVQLISIVVFTCACMNIEAGLVSRAPPTPAPVPPPGNKPPTLDSTKLKPGPGSAGGGTKFPPLSEAEKASLIEKVK<br>AKAAAGGTPGTTTTSPGGPPIKSETSKTAKTTKRAETPGAPGALGLDATKTKSAPSSTGGKIKTLDPRNNKPPQRHSAEE<br>VRAFMDKYGIKDSTVSPNFGISNTTPTGTGGYNSNITPTGTGGYNSNITPTGTGGYNSNTTPTGTGGYSKPSTGNLKSQ<br>TGYDRPPSNTTPTGTGGFIKPSTGNPKSQTGYDKPPPISTTNPTGTGGNVKPLTGNSKSQTGNDKPPPISTTNPTGTGG<br>NVKPLTGNSKSQTGNDKPTPISTPNQKPAHSPNGNHKSPPTNGKPPPAKAAGITKRTDSPMRNVLWTRSETPERRGQ
>PSTR_31_14585_12<br>MVSYKLAQLISIVVFTCACMNIEAGLVSRAAPSSTHVVAPGKPATLDSTKLQPVPGSAGGGGKLPPLSEAEKASLIEKVKA<br>KAAAGGTPGASLGGPPIKSEATKTAKTTKRAETGKPSGNQPTVDPTKPKTGTSPTGGNQKIPGSGNNKPTQKHPEEFK<br>AFMDKHGIKDSNASPIGGNSNANPTGGKGSSKPPAPKSPAPGTGGSNPNGR
>PSTR_31_14974_1<br>MTQLKFLLGLFGSLLATSVLGAGISDEDFAKLPEGMHIIKADKAGLPYVDPVTNEKFQNIQNKLDKEIMIHNGRDSWIIEP<br>RQNVHLDYDPNHPYFLITDNETVLLTKDSYKDYVTDTAIERLKEKAGERPTASPPTGCKDTSDYQHEEWYENLTPDAVQ<br>NTEEIADKSLPIIEGESQRDQSRESSRATGSGGEQKPQDAPMKEIQNAPSSRPVEPMIPVEPIHRGIRTGRWSKQRRPIIL<br>LHPQSSTFTPYMPFHTPHYFPHYPQFDGAVMKRIDPYTKQEYYEPTSYIYNAYPHR
>PSTR_31_15706_1<br>MGVPLLPIWLLLTGLLGRIGEGTCLSIYKDTLELATRDTDLLVSQREWATTILGGQRPPPEVKYAQMMTWKHPNTDESK<br>GKASDVRTGANHATLAEASFLSVSNQRT
>PSTR_31_15792_2<br>MLAPSHYLALVLTLLTGAWLARTTLAQILYDQPENTLDRLSTIFAKLAGNCTIIVSNPNKVEVSKHQIEDGYASIFNQCQP<br>NKTEVQHKTFFQKSGQTPLFDGVYLQSQNHSSGHDTDYFPPQTLTCGLNTNAPLTVGEDCQDAFESIFVDNKDRMVD<br>DNFQPTSSITKTRQTCTVLIYTTDNSPIVLKKSDISPVVLKTIEDCEGKSGVVSSTEGGSGYNGFTVVKVRSSKRCGSRSDSE<br>GQVCY
>PSTR_Yr9_00029_4<br>MPSTHITRAAVIGVFANVIQVSSLPAITPASAAAQPASASSEEMMTSMPTSTNRAAAVAPTTPSTTLSSAEVPMSPPTSS<br>SPASPTATIPKTVSPSGVVPAAAVVENTTAPATIKPPVSTNISAAQPNAASALPVTQAPGTTASVAPTTSPVSTITAPVTP<br>TTAPASAITAPITSTTVPVEAVDTTSRTVTTASKPAIAAPVETFSAGNSSSTANQAARNAVASSSSDSTDASSSANSQSIF<br>WAASHHNIFSIAHLHKAKNVYPSTPLKTSHAHMLAQSGAV
>PSTR_Yr9_00877_2<br>MLQLSGLMACVLLLLVAAPWEATSQEPVKIWFGCNKNVDALCVDKVDKDHRQLTFAERRKPHTRDYACWATLTPYCC<br>WQGKFKLWEKTWLWSPARITVARHV

>PSTR_Yr9_05355_3<br>MKLCEMLFLLGVILAEGAFVAANFGCHVDKRFTKGWCALPVTTDPATEKVLTYQMARGAVRSSGAFACPDDSRLVFSC<br>CGATFDVSSTQIIDFNVYGSNCDPHGLWVDKSWVNKELTARHKLFIFASRTRRDDPLCYLPPSATIAS
>PSTR_Yr9_05564_4<br>MNWLQLAILPVVICYFNIMMAATALREEMLNPVLDDHAIFANLDDIPEGNLEPKRRRIAEDHSDVIDSNPDSPNSSADS<br>FDTLMSSSLSGFSDHPHNPGSTVDRESWEMDTPHKTEAHRVPSSWGSGGSTKASIEAQGEMIQALREHFRQSKVLVE<br>AKQRSQRNSGISRSTNERTGFNWPKDPIPLKDEVGFEENQFTTAGKRKRPYGSDEGPIAGRENRIDAQKAEIAELYNIGK<br>LKQVERVRFGGLSMDMITGSKICVFLPMRNIQGWVWCRGKGPVMLPLF
>PSTR_Yr9_07161_2<br>MHHSFFTTALLIVSNVMAQDPSLSTTGLAPGVAPGVAPGSSGSTTSPTPGTGTGGGSGNGGGSGNGGGVGNGGGSG<br>NGGGVGNGGGSGNGGGSGNGGGVGNGGGAGNGGNNGNGGAPVTPTPPNGPKPIRVVCANSNLPFSERELAEMA<br>AMDTSSNSTQPTPDPKTLSEVQTAAICKNADLTAVCVLNSCNITTAPAPVCQSCYEYTPNPTGDDGIVGTVLTPQVTCE<br>DSYNFNQTDVKTPWVCSDKKQKTFSCGSCAGAR
>PSTR_Yr9_07312_1<br>MAKLSQLALNCALLIIFLVGTSSSAPDYSSLLTRDVRPDFDCHRTVADCEKRVPANKFSISSCHTSAQRVEQELQVPAAVK<br>EVVPKSSKASLACDFKTGTTSYKNGRIDLESKKAKNVSRNRHPDLLQIMTATSTSTR
>PSTR_Yr9_07780_2<br>MIMPLSRSGAGFWYPVLMLTIFVFRMVDAHAGIIKPPHLEPGAEFRRQCGQQAFGIVKGAPFISQESWKKRSRQCKDR<br>ICQGLFVKDQNLAAVPNFAPGAKIPVSIDVQIPHRCQAEAVAQSSCL
>PSTR_Yr9_08560_4<br>MNSSSFLTSLSVAFLIQAQILLVQAFLCTDSNVVSLGQTQPVCAIFFDGTHKALADQEGEPPSAPPGSTGQSGGGTGGG<br>TGGGSAGGKGSGTGKGSGTGKGSGTGKGSGGKGESDPFAYHIGKPTVVKDKHNKVIDYLCKPRHQTCCEQGIRVGHD<br>ISYKTWELKCTAMY
>PSTR_Yr9_08691_3<br>MLGQIQFLLLATLITPQFTFVVLGWTLPHTNTDFPSAVNERRSLIFKNGCNCRRIMVDLKVELTSPFGVKTRKFLLLHTGT<br>VFRPIFDSREEMEMVVEEKAEDGARAAIKDAKSGDAQVNRALGIVGTDGKKLLAGLEKLKDVAANPNSSKEQINSQLA<br>AVNKLREPLLGGCYVVGHKNFKKEH
>PSTR_Yr9_10469_2<br>MHLSSLLPIVALLLIQGGVTYVQSWGCKDAKGYNFASCVHVYPDLAVVQQGGKRVHWHVQQVIPPWDVNTYNCHK<br>GLVGYGIESCCYLESDSHGEAIMSYWTDRCRNKDGTPITPPY
>PSTR_Yr9_10579_3<br>MAVLATPSLLLLLLSPLLPVTAQDKLVDCKYSSPQGASYDFNNLIDPKSWPLNLTTITSTPPSTTIETILVSICKKLPTDHQT<br>QNPPTSCPDRTLVCLLIYNEIAGGLDRRLEQIIPVGLDTLSTPTYIALRQIGNQGEQNFLNLAKEKVT
>PSTR_Yr9_11114_1<br>MRVSCLSFPSVPLLSITLWLHPHTCAASLPFPALLNSTSAPYGIMQLADRLLPQPELPYHPVLRIFPRSTPLIPATAPIQPPL<br>PPSSTTFPPSCLPAIAVPRRVSTNPAPLPELPLMPLRNTFDGNTSAYQAAMIVWFDNRRVIQSYLNIPFNQPMPALEESA<br>AIQFSQRAAPATPVIDIDDIQTPVGSPINKGKERVVSPRPPVDCQERSNARVNSLTPTGVELLFGDARGGWLLREYVLM<br>YMYYVNNGVGHKPLALPSSPVGKNDYYGVPYNCPPPTFNSPAPPARPNTPVNPLHNLFPDCPQFARRYMPALIENKFL<br>RYIVALQWSAFTPSDSRNRYELFHSNCQLLRNATDDVILTKCIDSVEKCRYQRIGVTVQETLVSLVLVVQ

>PSTR_Yr9_11294_5<br>MKLFHFLGSSLLLGSGSAARIGLTAQYFQVDGSDENSIKLSWPADPTVHKFSVIRQSAGTSSSVIAQVQGNIYDDYGVPE<br>GELTYQVLVEGDPSKVSDQVTISSQPPFDSASSFSIYDNTQASNLKTIANIKLESTYYHFNIENDHDGVTQIIETTSTDGYTF<br>SGTKRVLLTRKELCSGSPDGFCKLESASFVQNPKNSEVVMWAHWEKGGPDYGQARVAVAFGQPGGEWEFGGSFRPL<br>ENQSRDLTFFHDNDGSGYLISSTAMNTNLNIYKLTPDWHNVTSLVSTVLKGQRREAPSLINHENVYYLFTSQASDWYPS<br>AGQYISATSLSGPWSQSRNIGNLAGFGAQSGSVQKIGSSWVMCANQWSGQWLDPEPPSHRVILPISLSNGYADYHYY<br>HRLRYDEAGIYGVQDGKVISVGQKCSSASASVPGFEESKSINGVNLDPNNFYLSATVPFSYEIEFEVASILSRFDLTTKLVG<br>GSETAYQFTISGRVSSTSLFRVILNKSNNTRVGFISSKVTDLTTYSAVRLDVHQVLNVHNKKQASWAQGIGQFTVYGHH<br>HQNSPTKSPDLKSARFSKRLLVGR
>PSTR_Yr9_11503_2<br>MFSYKFVLLISIVVFTCACMNIEAGLVSRAPPTPAPVPPPGNKPPTLDSTKLKPGPGSAGGGTKFPPLSEAEKASLIEKVKA<br>KAAAGGTPGTTTTSPGGPPIKSETSKMAKTTKRAETPGAPEALGLDATKTKSAPSSTGGKIKTLDPRNNKPPQRHSAEEV<br>RAFMDKYGIKDSTVSPNFGISNTTPTGTGGYNSNITPTGTGGYNSNITPTGTGGYNSNTTPTGTGGYSKPSTGNLKSQT<br>GYDRPPSNTTPTGTGGFIKPSTGNPKSQTGYDKPPPISTTNPTGTGGNVKPLTGNSKSQTGNDKPPPISTTNPTGTGGN<br>AKPLTGNSKSQTGNDKPTPISTPNQKPAHSPNGNHKSPPTNGKPPPAKAAGITKRTDSPMRNKDVVNKGLDGTKAGL<br>KEKMESMKSKMGGEGLAVASPGSGKPAPKATGGSKPIGQ
>PSTR_Yr9_11517_11<br>MVSYKLAQLISIVVFTCACMNIEAGLVSRAAPSSTHVVAPGKPATLDSTKLQPVPGSAGGGGKLPPLSEAEKASLIEKVKA<br>KAAAGGTPGASPGGPPIKSEATKTAKITKRAETGKPSGNQPTVDPTKPKTGTSPTGGNQKIPGSGNNKPTQKHPEEFKA<br>FMDKHGIKDSNASPIGGNSNANPTGGKGSSKPPAPKSPAPGTGGSNPNGR
>PSTR_Yr9_11853_1<br>MTQLKFLLGLFGSLLATSVLGAGISDEDFAKLPEGMHIIKADKAGLPYVDPVTNEKFQNIQNKLDKEIMIHNGRDSWIIEP<br>RQNVHLDYDPNHPYFLITDNETVLLTKDSYKDYVTDTAIERLKEKAGERPTASPPTGCKDTSDYQHEEWYENLTPDAVQ<br>NTEEIADKSLPIIEGESQRDQSRESSRATGSGGEQKPQDAPMKEIQNAPSSRPVEPMIPVEPIHRGIRTGRWSKQRRPIIL<br>LHPQSSTFTPYMPFHTPHYFPHYPQFDVPL
>PSTR_Yr9_12449_1<br>MGVPLLPIWLLLTGLLGRIGEGTCLSIYKDTLELATRDTDLLVSQREWATTILGGQRPPPEVKYAQMMTWKHPNTDESK<br>GKASDVRTGANHATQNLKQLEGKVFLHLEKLTHYPLGQGFAEGTGEKYSEHMMRLRSDVMELTPEMDETTSTPDLLY<br>GLHQENHDPG
>PSTR_Yr9_12515_2<br>MLAPSHYLALVLTLLTGAWLARTTLAQILYDQPENTLDRLSTIFAKLAGNCTIIVSNPNKVEVSKQQIEDGYASIFNQCQP<br>NKTEVQHKTFFQKSGQTPLFDGVYLQSQNHSSGHDTDYFPPQTLTCGLNTNAPLTVGEDCQDAFESIFVDNKDRMVD<br>DNFQPTSSITKTRQTCTVLIYTTDNSPIVLKKSDISPVVLKTIEDCEGKSGVVSSTEGGSGYNGFTVVKVRSSKRCGSRSDSE<br>GQVCY
>PSTR_K_00038_4<br>MPSTHITRAAVIGVFANVIQVSSLPAITPASAAAQPASASSEEMMTSMPTSTNRAAAVAPTTPSTTLSSAEVPMSPPTSS<br>SPASPTATIPKTVSPSGVVPAAAVVENTTAPATIKPSVSTNISSAQPNAASALPVTQAPGTTASVAPTTSPVSTITAPVTPT<br>TAPASAITAPITSTTVPVEAVDTTSRTVTTASKPAIAAPVETFSAGNSSSTANQAARNAVASSSSDSTDASSSANSQSIFW<br>AASPRRYIQPQLTRSANPKLQLFKGII
>PSTR_K_01210_2<br>MLQLSGLMACVLLLLVAAPWEATSQEPVKIWFGCNKNVDALCVDKVDKDHRQLTFAERRKPHTRDYACWATLTPYCC<br>WQGKFKLWEDPGWTRRVKTKELDDCKEGGQ
>PSTR_K_07248_3<br>MKLCEMLFLLGVILAEGAFVAANFGCHVDKRFTKGWCALPVTTDPATEKVLTYQMARGAVRSSGAFACPDDSRLVFSC<br>CGATFDVSSTQIIDFNVYGSNCDPHGLWVDKSWVNKELTARHKLFIFASRTRRDDPLCYLPPSATIAS

>PSTR_K_07556_4<br>MNWLQLAILPVVICYFNIMMAATALREEMLNPVLDDHTIFANLDDIPEGNLEPKRRRIAEDHSDVIDSNPDSPNSSADS<br>FDTLMSSSLSGFSDHPHNPGSTVDRESWEMDTPHKTEAHRVPSSWGSGGSTKASIEAQGEMIQALREHFRQSKVLVE<br>AKQRSQRNSGISRSTNERTGFNWPKDPTPLKDEVGFEENRFTTTGERKRPYGSNEGPIAGRENRIDAQKAEIAELYNMG<br>KLKQVERNWRGKQTGG
>PSTR_K_09855_2<br>MHHSFFTTALLIVSNVMAQDPSLSTTGLAPGVAPGVAPGSSGSTTSPTPGTGTGGGAGNGGGSGNGGGVGNGGGV<br>GNGGGVGNGGGSGNGGGSGNGGGVGNGGGAGNGGNNGNGGAPVTPTPPNGPKPIRVVCANSNLPFSERELAEM<br>AAMDTSSNSTQPTPDPKTCKYTYATPQRHLSSPRDSLPSVSEVQTAAICKNADLTAVCVLNSCNITTAPAPVCQSCYEYT<br>PNPTGDDGIVGTVLTPQVTCEDSYNFNQTDVKTPWVCSDKKQKTFSCGSCAGARACDICYDVKDIPTNPPTTNPPTTN<br>PPTTTPTTPTPSTSPPTPITPSLPATSNTPTTPNPVNPPTTAPATS
>PSTR_K_10081_1<br>MAKLSQLALNCALLIIFLVGTSSSAPDYSSLLTRDVRPDFDCHRTVADCEKRVPANKSISLSCKVECHKVSFVGLTSVNRPS<br>YRADSQAFNISFRCSNRTLVGSLSF
>PSTR_K_10703_1<br>MIMPLSRSGAGFWYPVLMLTIFVFRMVDAHAGIIKPPHLEPGAEFRRQCGQQAFGIVKGAPFISQESWKKRSRQCKDR<br>ICQGLFVKDQNLAAVPNFAPGAKIPVSIDVQIPHGGVARLLLVDMKNEKTFQSGGKDIVLQDMVEIVTATLPTVTPKEC<br>SEPGRCAISSTKEKATIFVL
>PSTR_K_11743_4<br>MNSSSFLKSLSVAFLIQAQILLVQAFLCTDSNVVSLGQTQPVCAIFFDGTHKALADQEGEPPSAPPGSTGQSGGGTGGG<br>TGGGSAGGKGSGTGKGSGTGKGSGTGKGNGGKGESDPFAYHIGKPTVVKDKHNKVIDYLCKPRHQTCCEQPPVLISL<br>WRS
>PSTR_K_11954_3<br>MLGQIQFLLLATLITPQFTFVVLGWTLPHTNTDFPSAVNERRSLIFKNGCNCRRIMVDLKVELTSPFGVKTRKFLLLYTGT<br>VFRPIFDSREENGNGGGGRGGGRTTKTSPQPKPSATGAKKTSLLSVGNPTTTRGKPVNPGQRGRGGQAGSQLSQLSV<br>QPGKPGGKNPGRQAAVKLDQQVKQVTEGLKSIPGKSGPQVVREVNRILGIEKAEDGARAAIKDAKSGDAQVNKALGIV<br>GTDGKKLLAGLEKLKDVAANPNSSKEQINSQLAAVNKLREPLLGGCYVVGHKNFKKEH
>PSTR_K_14315_3<br>MHLSSLLQILVVILIQGGVTHVQSWGCEKAGNDYRSAGCIYILPDLNPPSFGQKPRPWHLKLMISPWDNVKKTYSCDKA<br>PRGFKTQACCTSDKVLHDGTTTGIFVGICKKLDGTEFKF
>PSTR_K_14449_2<br>MAVLVTLSLLLLLLSPLLLVTAQDKLVDCKYSSPQGASYDFNNLIDPKSWPLNLTTITSTPPSTTIETILVSICKKLPTDHQTQ<br>NPPTSCPDRTLVCLLIYNEIAAGLDRRLEQIIPVGLDTLPTPTYIALRQIGNQVKKLCLKRSLASQREPYMIGYKGL
>PSTR_K_15086_1<br>MRVSCLSFPLVPLLSITLWLHPHTCAASLPFPALLNSTSAPYGIMQLADRLLPQPELPYHPVLRIFPRSTPLIPATAPIQPPL<br>PPSSTTFPPSCLPAIAVPRRVSTNPAPLPELPLMPLRNTFDGNTSAYQAAMIVWFDNRWVIQSYLNIPFNQPMPALEES<br>AAIQFSQRAAPATPVIDIDDIQTPVGSPITACVKDSPPPMLRSIC
>PSTR_K_15401_5<br>MKLFHFLGSSLLLGSGSAARIGLTAQYFQVDGSDENSIKLSWPADPTVHKFSVIRQSAGTSSSVIAQVQGNIYDDYGVPE<br>GELTYQVLVEGDPSKVSDQVTISSQPSFDSASSLSIYDNTQASNLKAIANIKLESTYYHFNIENDHDGVTQIIETTSTDGYTF<br>SGTKRVLLTRKELCSGSPDGFCKLESASFVQNPKNSEVVMWAHWEKGGPDYGQARVAVAFGQPGGEWEFGGSFRPL<br>ENQSRDLTFFHDNDGSGYLISSTAMNTNLNIYKLTPDWHNVTSLVSTVLKGQRREAPSLINHENVYYLFTSQASDWYPS<br>AGQYISATSLSGPWSQSRNIGNLAGFGAQSGSVQKIGSSWVMCANQWSGQWLDPEPPSHRVILPISLSNGYADYHYY<br>HRLRYSEAGIYGVQDGKVISVGQKCSSASASVPGFEESKSINGVNLDPNNFYLSATVPFSYEIEFEVASILSRFDLTTKLVG<br>GSETAYQFTISGRVSSTSLFRVILNKSNNTRVGFISSKVTDLTTYSAVRLDVHQVLNVHNKKQASWAQGIGQFTVYGHH<br>HQNSPTKSPDLKSARFSKRLLVGR

>PSTR_K_15644_3<br>MFSYKFVLLISIVVFTCACMNIEAGLVSRAPPTPAPVPPPGNKPPTLDSTKLKPGPGSAGGGTKFPPLSEAEKASLIEKVKA<br>KAAAGGTPGTTTTSPGGPPIKSETSKTAKTTKRAETPGAPEALGLDATKTKSAPSSTGGKIKTLDPRNNKPPQRHSAEEV<br>RAFMDKYGIKDSTVSPNFGISNTTPTGTGGYNSNITPTGTGGYNSNITPPGTGGYNSNTTPTGTGGYSKPSTGNLKSQT<br>GYDKPPSNTTPTGTGGFIKPSTGNPKSQTGYDKPPPISTTNPTGTGGNVKPLTGNSKSQTGNDKPPPISTTNPTGTGGN<br>VKPLTGNSKSQTGNDKPTPISTPNQKPAHSPNGNHKSPPTNGKPPPAKAAGITKRTDSPMRNKDVVNKGLDGTKAGL<br>KEKMESMKSKMGGEGLAVASPGLENPLPRPPVAASQLGNDNCLCCLTYIHPIPDLSLHK
>PSTR_K_15672_11<br>MVSYKLAQLISIVVFTCACMNIEAGLVSRAAPSSTHVVAPGKPATLDSTKLQPVPGSAGGGGKLPPLSEAEKASLIEKVKA<br>KAAAGGTPGASLGGPPIKSEATKTAKITKRAETGKPSGNQPTVDPTKPKTGTSPTGGNQKIPGSGNNKPTQKHPEEFKA<br>FMDKHGIKDSNASPIGGNSNANPTGGKGSSKPPAMKSPAPGTGGSNPNGR
>PSTR_K_16133_2<br>MTQLKFLLGLFGSLLATSVLGAGISDEDFAKLPEGMHIIKADKAGLPYVDPVTNEKFQNIQNKLDKEIMIHNGRDSWIIEP<br>RQNVHLDYDPNHPYFLITDNETVLLTKDSYKDYVTDTAIERLKEKAGERPTASPPTGCKDTSDYQHEEWYENLTPDAVQ<br>NTEEIADKSLPIIEGESQRDQSRESSRATGSGGEQKPQDAPMKEIQNAPSSRPVEPMIPVEPIHRGIRTGRWSKQRRPIIL<br>LHPQSSTFTPYMPFHTPHYFPHYPQFDGAVMKRIDPYTKQEYYEPTSYIYNAYPNR
>PSTR_K_17012_1<br>MGVPLLPIWLLLTGLLGRTGEGTCLSIYKDTLELATRDTDLLVSQREWATTILGGQRPPPEVKYAQMMTWKHPNTDES<br>KGKASDVRTGANHPALAEASFLSVSNQRT
>PSTR_K_17117_2<br>MLAPSHYLALVLTLLTGAWLARTTLAQILYDQPENTLDRLSTIFAKLAGNCTIIVSNPNKVEVSKHQIEDGYASIFNQCQP<br>NKTEVQHKTFFQKSGQTPLFDGVYLQSQNHSSGHDTDYFPPQTLTCGLNTNAPLTVGEDCQDAFESIFVDNKDRMVD<br>DNFQPTSSITKTRQTCTVISGSLTVKLKISGPLKPIFSSPKKIPSEYTSKKSDISPVVLKTIEDCEGKSGVVSSTEGGSGYNGFT<br>VVKVRSSKRCGSRSDSEGQVCY

Supplementary Table 21 Comparative presentation of genes with dN/dS> 1 for three *P. striiformis* genomes across the extracellular proteins

Gene ID	Length (aa)	dN/dS	Functional Annotation
PSTR_31_01029_3	161	1.20155	hypothetical protein
PSTR_31_06103_4	398	1.6309	Not annotated
PSTR_31_09456_1	105	1.7637	Not annotated
PSTR_31_16356_1	370	1.4972	Not annotated
PSTR_K_06650_4	398	1.6309	Not annotated
PSTR_K_17760_1	370	1.6361	Not annotated
PSTR_Yr9_02181_1	278	1.1254	Not annotated
PSTR_Yr9_06473_1	116	1.43185	Not annotated
PSTR_Yr9_07542_3	115	1.7637	Not annotated
PSTR_Yr9_12972_1	370	1.4972	Not annotated

*Yr9 refers to P.str 46S 119 pathotype

Supplementary Table S22 fasta sequence of 10 genes with *dN/dS* ratio above 1 by *YN00*

>PSTR_31_01029_3<br>MQKFIFVTSLWMISLIAGTGGAMLSGFHTSREGTTASKPMKWEPESRYYHRDADGIETCKKIDALKSGTTVQLLARSGK<br>GAQEFHKVDTGGVSGGFQFNSKKSGFLTLLSIGNSDIVYILHDVKAKAVVWEKFLKRGATHHFSFPSGADDGDIQLYTRI<br>A
>PSTR_31_06103_4<br>MKATFSLPLLLLLQISTSLLFNLASSPTQARVEAVDVQPILSVDRQEDEIHQYLSDPLSFPETTLPLKNPNPTFHLPSTPHEL<br>PSQSPTDGFYSKGDHTTNVLLPSNRFFPSGSSGLIPSTEVHRSHSDLTTGDANCRSDITYTGDLTSSRRPTSDSDLICYRKD<br>LACKRKGQELIRQNEPCVPLVRKRVKVTQGQNDQKSRDRAIELLLSISDFNPNELQDIDKGTAKPPGSLAQSDNPYDRR<br>QGSRKFVAGAPEDVANEFPQDEKTRSDQDSLHDNLLEGLANSVDTRNPISAKNTRPGGTRRPDIGETLLALSDATIEIEK<br>YDLFVQIMNLRKNQISEWASRQQNGGNACRTRDRILKFVKSITKMSTLLIKGITRDITSTSLVAQDINDVGSK
>PSTR_31_09456_1<br>MVVFLLQLRASLVNSIDCSKAGEKNQHAGCGKYVEQKTIPGHTDAPYWTIDVIPPPWYQDTYDCKQSKGYTIEACCSVS<br>PRFIFSTSRTSGLCVKPDGSALKLK
>PSTR_31_16356_1<br>MDNGIVWVLVHCWYKRHPMDPHSVAVFYRMKLFLADLNHVLGAVIPFKHQLNCNHANFQNRSSDCRPTHQSRAW<br>KRSWLPQLKTQIEGFKSQHTPKTCVVQLPSRTRPIESMYKEHLPNFDTPDVSQRESQYCQAPHYQYTAQPKQQYLEEPY<br>TKEPDQQYSEQSYACGHYQQHLGREDYSERCSKGPADGSYIDGDSAVIEDVVSNYHSDSSKNCNNASVGHVFEHELPS<br>DPDPESSDTTLQEAHPYIVQPDQQEPDFDMANCSATDIDCWAITCNDQDEYIDYKPDNTDDAVPHCFETGDGDQDPY<br>KTSNDSFHNGCDGGNGLDHSHEASLEDLNCNDVLNGDSNNFDGSHVILLLLLRVFHVSTCY
>PSTR_Yr9_02181_1<br>MHHTFLAAALLVFTNVLAQDPSPLYQWRCTRHQRRKYRRLKLKLYHHTEHPQPCARRPKPISVKCTNTHLPFSKRELAE<br>MAQMDASSGTAGNTVPATPDPKTRLPPSVSDVQTAAICKNDLVTAVCELHSCNVTTSPPPVCQSCYEFTPNPNGDEGI<br>VGTVLTQQVICEDSYNFNKTDVKTPNVCSDKNQKTFSCGSCTGERACDVCYDVKDIPTNPNTTPNPSGPTPPTTPTTPT<br>TPPTTTTATTPSPPTTSNTPTTPTTPPTNP
>PSTR_Yr9_06473_1<br>MNLLPLVVLLSCAITLAVLADGNGAKPDPAKATKVVFKCSDAKELTAGWCVSNVPGADKRSFVKANVVGPPKDLNYNC<br>IDTNKENNMCCKADFKPDQKGEGSPGTDVCVIKGPVP
>PSTR_Yr9_07542_3<br>MKLSNFPGFPMVLLILQLKASLVDSMDCSKAGEKNQHAGCGKYVEQKTIPGHTDAPYWTIDVIPPPWYQDTYDCKQS<br>KGYTIEACCSVSPRFIFSTSRTSGLCVKPDGSALKLK
>PSTR_Yr9_12972_1<br>MDNGIVWVLAHCWYKRHPMDPHSVAVFYRMKLFLADLNHVLGAVIPFKHQLNCNHANFQNRSSDCRPTHQSRAW<br>KRSWLPQLKTQIEGFKSQHTPKTCVVQLPSRTRPIESMYKEHLPNFDTPDVSQRESQYCQAPHYQYTAQPKQQYLEEPY<br>TKEPDQQYSEQSYACGHYQQHLGREDYSERCSKGPADGSYIDGDLAFIKDVVSNYHSDLSKNCNNASVGHVFERKLPS<br>DPDPESSDTTLQEAHPYIVQPDQQEPDFDMANCSATDIDCWAITCNDQDEYIDYKPDNTDDAVPHCFETGDGDQDPY<br>ETSNGSFHNGCNGGNGLDHSHEASLEDLNCNDVLNGDSNNFDGSHVILLLLLRVFHVSTCY
>PSTR_K_06650_4<br>MKATFSLPLLLLLQISTSLLFNLASSPTQARVEAVDVQPILSVDRQEDEIHQYLSDPLSFPETTLPLKNPNPTFHLPSTPHEL<br>PSQSPTDGFYSKGDHTTNVLLPSNGFFPSGSSGLIPSTEVHRSHSDLTTGDANCRSDITYTGDLTSSRRPTSDSDLICYRKD<br>LACKRKGQELIRQNEPCVPLVRKRVKVTQGQNDQKSRDRAIELLLSISDFNPNELQDIDKGTAKPPGSLAQSDNPYDRR<br>QGSRKFVAGAPEDVANEFPQDEKTRSDQDSLHDNLLEGLANSVDTRNPISAKNTRPGGTRRPDIGETLLALSDATIEIEK<br>YDLFVQIMNLRKNQISEWTSRQQNGGNACRTRDRILKFVKSITKMSTLLIKGITRDITSTSLVAQDINDVGSK

>PSTR_K_17760_1
MDNGIVWVLAHCWYKRHPMDPHSVAVFYRMKLFLADLNHVLGAVIPFKHQLNCNHANFQNRSSDCRPTHQSRAW
KRSWLPQLKTQIEGFKSQHTPKTCVVQLPSRTRPIESMYKEHLPNFDTPDVSQRESQYCQAPHYQYTAQPKQQYLEEPY
TKEPDQQYSEQSYACGHYQQHLGREDYSERCSKGPADGSYIDGDSAVIEDVVSNYHSDSSKNCNNASVGHVFERKLPS
DPDPESSDTTLQEAHPYIVQPDQQEPDFDMANCSATDIDCWAITCNDQDEYIDYKPDNTDDAVPHCFETGDGDQDPY
KTSNGSFHNGCNGGNGLDHSHEASLEELNCNDVLNGDSNNFDGSHVILLLLLRVFHVSTCY

Supplementary Table S23 Identification of extracellular proteins of *P. striiformis* genomes under purifying selection

	dN/dS (YN00) >0 and <1	dN/dS (YN00)=0	dN/dS (YN00) >1
P. *str* 31	53	290	4
P. *str* K	35	304	2
P. *str* 46S119.	89	236	4

Supplementary Table S24 Categorization of genes according to the dN/dS obtained

	dN/dS (YN00) >0 and <1	dN/dS (YN00)=0	dN/dS (YN00) >1
P.str 31	53	290	4
P.str K	35	304	2
P.str 46S119	89	236	4

Supplementary Table S25 Comparative analysis of different *P. striiformis* genomes

	Year	Origin	Genome Size (Mb)	Repeats Content (Mb)	Repeats as % of genome	Largest Contig (Kb)	Contigs (K)
P.str 31	1936	INDIA	66.26	24.39	36.80	54	30.066
P.str K	1982	INDIA	69.77	25.31	36.27	67	32.818
P.str 46S 119	1996-2000	INDIA	70.24	24.75	35.23	73	24.737
P.str 21	1980	U.S.	73.05	30.04	41.12	37	43.101
P.str 43	1990	U.S.	70.67	29.87	42.26	35	49.546
P.str 87-7	2003	U.K.	53.40	25.68	48.08	46	55.495
P.str 130	2007 or 2009	U.S.	64.78	25.30	39.05	49	29.178
P.str 8-21	2008	U.K.	56.27	26.35	46.82	35	50.890
P.str 78-1	2007	U.S.	79.31	24.65	31.08	109	17.295
P.str Cy32	2007	China	115.48	33.27	28.81	143	12.528
